# Design, Synthesis, and Evaluation of a New Series of 2-Pyrazolines as Potential Antileukemic Agents

**DOI:** 10.1021/acsomega.3c05860

**Published:** 2023-10-30

**Authors:** Mehlika Dilek Altıntop, Zerrin Cantürk, Ahmet Özdemir

**Affiliations:** † Department of Pharmaceutical Chemistry, Faculty of Pharmacy, Anadolu University, 26470 Eskişehir, Turkey; ‡ Department of Pharmaceutical Microbiology, Faculty of Pharmacy, Anadolu University, 26470 Eskişehir, Turkey

## Abstract

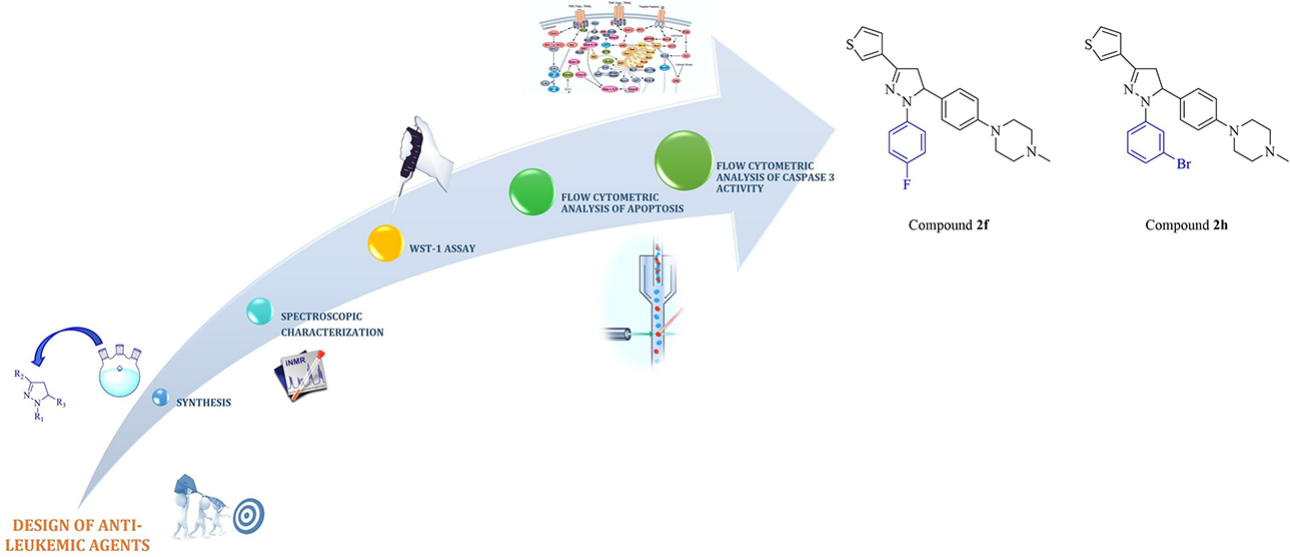

In an attempt to identify small molecules for the treatment of leukemia, 12 new pyrazolines (**2a**–**l**) were synthesized efficiently. WST-1 assay was performed to examine their cytotoxic features on HL-60 human acute promyelocytic leukemia (APL), K562 human chronic myeloid leukemia (CML), and THP-1 human acute monocytic leukemia cells. Four compounds (**2e**, **2f**, **2g**, and **2h**) were determined as promising antileukemic agents on HL-60 and K562 cells. IC_50_ values of compounds **2f**, **2h**, **2e**, **2g**, and bortezomib for the HL-60 cell line were found as 33.52, 42.89, 48.02, 62.34, and 31.75 μM, while IC_50_ values of compounds **2h**, **2g**, **2f**, **2e**, and bortezomib for K562 cells were determined as 33.61, 50.23, 57.28, 76.90, and 42.69 μM, respectively. Further studies were carried out to shed light on the mechanism of antileukemic action. According to the data obtained by *in vitro* experiments, 1-(4-fluorophenyl)-3-(thiophen-3-yl)-5-(4-(4-methylpiperazin-1-yl)phenyl)-2-pyrazoline (**2f**) and 1-(3-bromophenyl)-3-(thiophen-3-yl)-5-(4-(4-methylpiperazin-1-yl)phenyl)-2-pyrazoline (**2h**) have proved to be potential antileukemic agents with remarkable cytotoxicity against HL-60 and K562 cells by activation of caspase 3, thereby inducing apoptosis.

## 1 Introduction

Leukemia, the most common childhood cancer, is a category of hematological malignancies caused by the rapid and uncontrolled proliferation of aberrant white blood cells. Based on the origin and the clinical features of cells, leukemia is divided into four main types, namely, acute lymphocytic leukemia (ALL), chronic lymphocytic leukemia (CLL), acute myeloid leukemia (AML), and chronic myeloid leukemia (CML).^1−3^

Leukemia is typically treated with cytotoxic chemotherapy, radiation, and more recently, targeted therapy.^3^ Resistance to chemotherapeutics remains a major challenge in the fight against leukemia.^4^ Another issue that conventional chemotherapy faces is the lack of selectivity. Anticancer drugs, in general, damage not only cancer cells but also normal cells, and therefore, their use in cancer therapy frequently results in severe toxicity and adverse effects. As a result, small molecules endowed with selective antineoplastic activity are constantly being developed to selectively eliminate tumor cells or at the very least, inhibit their proliferation.^5^

**Figure 1:**
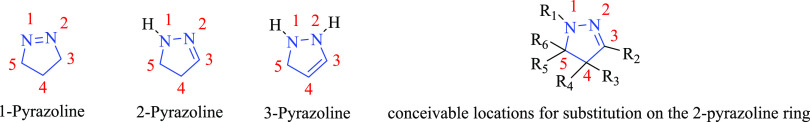
Pyrazolines.

Dihydropyrazoles, commonly known as pyrazolines, are nonaromatic five-membered heterocyclic compounds with two nitrogen atoms at neighboring locations (Figure 1). Pyrazoline, considered as a cyclic hydrazine motif, possesses an endocyclic double bond.^6,7^ Pyrazolines are stronger bases than pyrazoles. However, they are less stable and behave more like unsaturated compounds. Pyrazoline moiety is highly prevalent with lipophilic characteristics and soluble in most organic solvents.^8^ When aryl groups exist in positions 1 and 3, the 2-pyrazoline ring serves as an inner ring in a scintillation solute molecule.^6,8^ 2-Pyrazolines are privileged heterocyclics used in industries as medicines, biorelated materials, and so on.^9^ Diversely substituted pyrazolines have been reported to possess a broad range of pharmacological effects, particularly antitumor activity.^8−19^ Some pyrazoline-based cytotoxic agents also exert cancer chemopreventive action.^10^

Thiophene is a five-membered heteroaromatic ring containing a sulfur atom. Many researchers have also intensively examined thiophenes because novel useful compounds can be designed by bioisosteric replacement of the benzene with the thiophene.^20^ Several studies have confirmed that compounds containing the thiophene moiety have anticancer action.^11^

**Figure 2:**
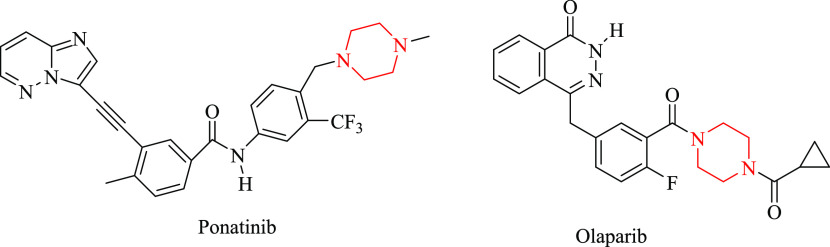
Nitrogen-containing heterocyclic antineoplastic agents approved by the FDA in the recent decade.

Piperazine is another common *N*-heterocyclic molecule that is regarded as one of the most vital building blocks of many major natural and synthetic anticancer agents.^21^ In recent years, the U.S. Food and Drug Administration (FDA) has approved a large number of nitrogen-containing heterocycles as chemotherapeutics. Among them, ponatinib and olaparib are nitrogen-containing heterocyclic agents currently used for the treatment of CML, Philadelphia chromosome-positive ALL, and advanced ovarian cancer, respectively (Figure 2).^22^

Taking into account the data concerning the anticancer effects of 2-pyrazolines, piperazines, and thiophenes, herein, we aim to synthesize a new series of small-molecule antileukemic agents carrying these important moieties on the same skeleton.

## 2 Results and Discussion

### 2.1 Chemistry

**Scheme 1:**
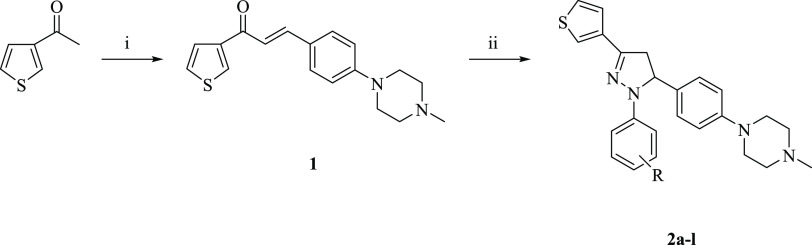
Synthesis of Compounds **2a**–**l** Reagents and conditions: (i) 4-(4-methylpiperazin-1-yl)benzaldehyde, 10% sodium hydroxide solution, ethanol, rt, 16 h; (ii) arylhydrazine hydrochloride, ethanol, reflux, 22 h.

In the present work, 3-[4-(4-methylpiperazin-1-yl)phenyl]-1-(thiophen-3-yl)prop-2-en-1-one (**1**) was synthesized as described earlier^23^ and treated with arylhydrazine hydrochloride derivatives to obtain compounds **2a**–**l** (Scheme 1).

**Figure 3:**
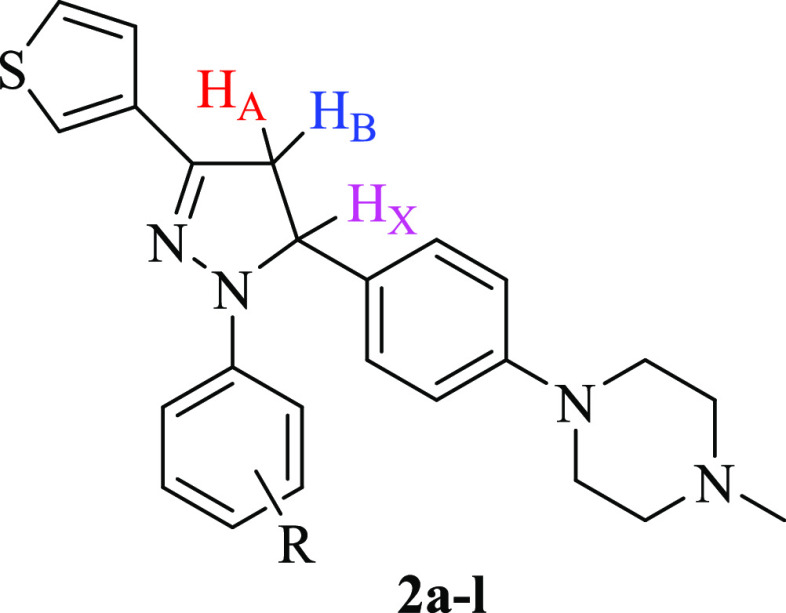
ABX pattern of the pyrazoline scaffold.

The structures of the newly synthesized compounds were elucidated by infrared (IR), nuclear magnetic resonance (NMR, ^1^H and ^13^C), and high-resolution mass spectrometry (HRMS). The absence of the C= O stretching band at 1645.28 cm^–1^ in the IR spectra of compounds **2a**–**l** revealed that ring closure occurred efficiently. In the ^1^H NMR spectra of all compounds, the CH_2_ protons of the pyrazoline core resonated as a pair of doublets at δ 2.95–3.13 and 3.72–3.92 ppm. The CH proton was observed as a doublet of doublets at δ 5.22–5.53 ppm due to the vicinal coupling with two magnetically nonequivalent protons of the methylene moiety at position 4 of the pyrazoline scaffold (*J*_AB_ = 17.31–17.79 Hz, *J*_AX_ = 4.62–6.90 Hz, *J*_BX_ = 11.40–12.12 Hz) (Figure 3). Moreover, the methyl protons attached to the piperazine ring appeared as a singlet peak at δ 2.62–2.98 ppm. In the ^1^H NMR spectra of compounds **2a**–**l**, the multiplets or the broad singlets in the region 3.19–3.40 ppm were attributed to the piperazine protons. The signals obtained from the ^13^C NMR spectra also confirmed the proposed structures. The C_4_ and C_5_ carbons of the pyrazoline ring resonated at 42.55–44.25 and 61.32–63.76 ppm, respectively. All compounds showed a signal at 149.00–154.58 ppm, which was assignable to the azomethine carbon of the 2-pyrazoline. In the ^13^C NMR spectra of compounds **2a**–**l**, the signals due to the C_2_ and C_6_ carbons of the piperazine ring were observed in the region 45.84–46.75 ppm, whereas the signals due to the C_3_ and C_5_ carbons of the piperazine core were detected in the region 52.60–53.62 ppm. The formation of the 2-pyrazoline scaffold was further confirmed by the HRMS data of compounds **2a**–**l**.

### 2.2 Anticancer Activity

WST-1 assay was performed to test the cytotoxic effects of compounds **2a**–**l** on HL-60 human acute promyelocytic leukemia (APL), K562 human CML, and THP-1 human acute monocytic leukemia cells (Table 1). Among them, compounds **2f**, **2h**, **2e**, and **2g** exerted marked anti-APL activity against the HL-60 cell line with IC_50_ values of 33.52, 42.89, 48.02, and 62.34 μM, respectively compared to bortezomib, the positive control (IC_50_ = 31.75 μM). The cytotoxic effect of compound **2f** on the HL-60 cell line was similar to that of bortezomib. Moreover, compounds **2h**, **2g**, **2f**, and **2e** were reported to show pronounced anti-CML effect on K562 cells with IC_50_ values of 33.61, 50.23, 57.28, and 76.90 μM, respectively, compared to bortezomib (IC_50_ = 42.69 μM). As presented in Table 1, compound **2h** was more potent on the K562 cell line than bortezomib. On the other hand, the IC_50_ data obtained by the WST-1 assay revealed that the tested compounds did not show significant activity toward THP-1 cell line. It can be concluded that *p*-fluoro substitution significantly enhances not only anti-APL but also anti-CML activity. On the contrary, *m*-fluoro substitution dramatically decreases both anti-APL and anti-CML activity. This outcome confirms the importance of the position of the fluoro group on the phenyl ring attached to the first position of the pyrazoline scaffold for antileukemic activity. A similar situation exists for *p*- and *m*-chlorine substitution. On the other hand, *p*-bromo and *m*-bromo substitutions caused similar anti-APL action, while *m*-bromo substitution increased anti-CML activity more than *p*-bromo substitution. 4-Cyano and 4-methoxy substitutions led to a significant decline in anti-APL activity.

**Table 1:** IC_50_ Data of Compounds **2a**–**l** and Bortezomib for HL-60, K562, and THP-1 Cells

		IC_50_ (μM)		
compound	R	HL-60 cell line	K562 cell line	THP-1 cell line
**2a**	H	149.47	277.84	460.24
**2b**	4-SO_2_CH_3_	149.25	132.60	408.97
**2c**	4-CH_3_	98.86	93.54	436.51
**2d**	4-CN	317.48	198.34	444.94
**2e**	4-Br	48.02	76.90	301.58
**2f**	4-F	33.52	57.28	390.68
**2g**	4-Cl	62.34	50.23	294.42
**2h**	3-Br	42.89	33.61	481.98
**2i**	3-Cl	186.32	111.78	339.09
**2j**	3-F	176.33	363.10	221.35
**2k**	3-NO_2_	143.95	141.31	194.69
**2l**	4-OCH_3_	321.75	209.13	287.03
**Bortezomib**		31.75	42.69	108.67

Controlling or possibly stopping the uncontrolled growth of cancer cells is a promising way for cancer therapy. An efficient route to accomplishing this goal is the cell’s inherent mechanism for death. As a result, targeting apoptosis, the most successful nonsurgical therapeutic strategy, is effective for all types of cancer since evasion of apoptosis is a crucial hallmark of cancer and is not specific to the etiology or type of cancer.^24^ Apoptosis is executed by cysteine-dependent aspartyl-specific proteases (caspases), a family of cysteine proteases. Among caspases, caspase 3 is a key executioner protein involved in proteolytic degradation during apoptosis.^25^ Caspase 3 activation leading to the induction of apoptosis is an outstanding approach for the management of cancer.^26^

**Figure 4:**
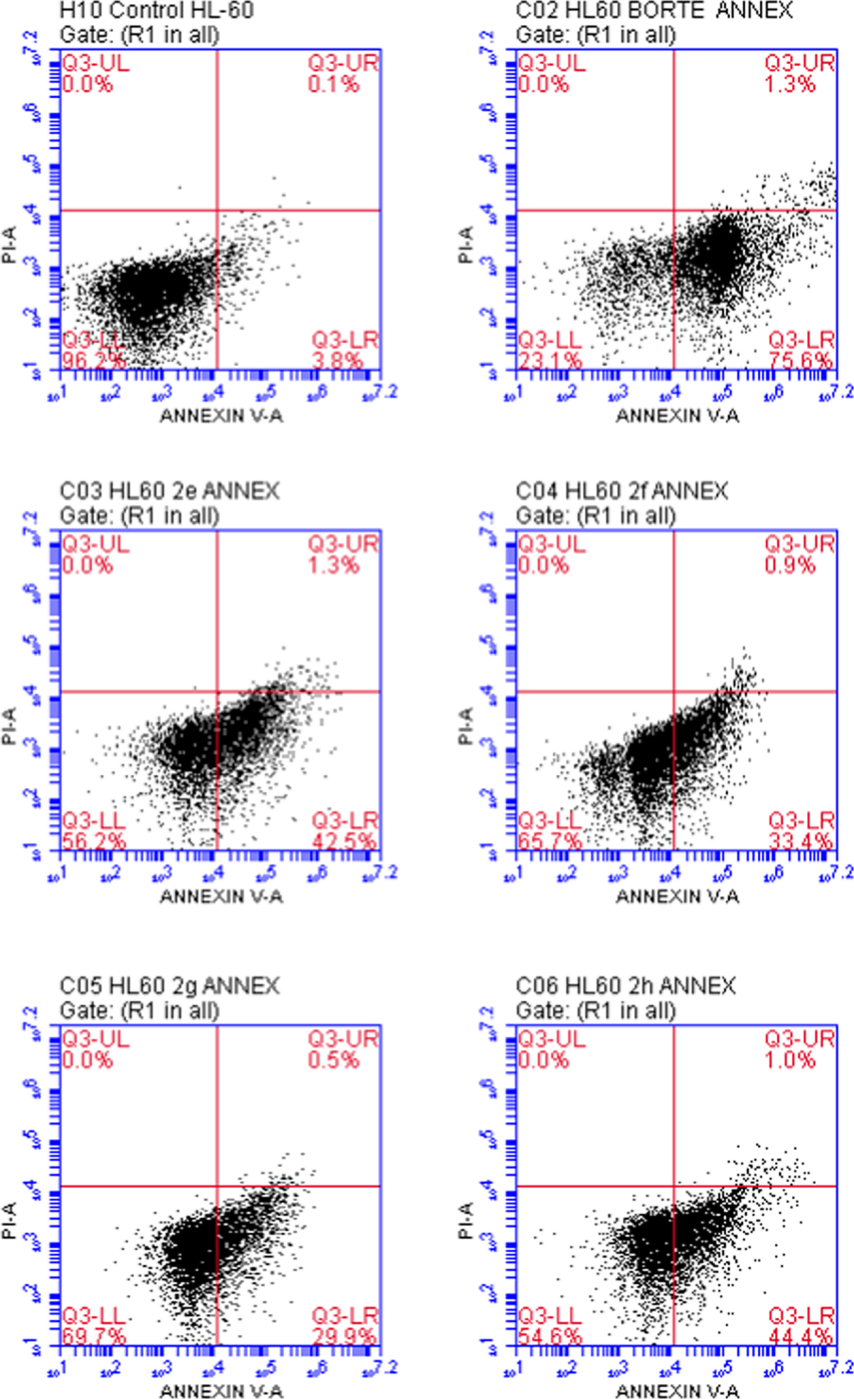
Flow cytometric analysis of HL-60 cells treated with IC_50_ values of compounds **2e**–**h** and bortezomib.

A flow cytometry-based apoptosis detection test was used to examine the effects of compounds **2e**–**h** and bortezomib on apoptosis utilizing Annexin V-fluorescein isothiocyanate (FITC)/propidium iodide (PI) staining in HL-60 APL and K562 CML cells after the 24 h incubation period. The findings demonstrated that HL-60 APL and K562 CML cells treated with the tested compounds underwent apoptosis. The percentages of HL-60 cells undergoing early apoptosis exposed to compounds **2e**, **2f**, **2g**, **2h**, and bortezomib at IC_50_ concentrations were found to be 42.5, 33.4, 29.9, 44.4, and 75.6%, respectively (Table 2, Figure 4). The percentages of HL-60 cells undergoing late apoptosis exposed to compounds **2e**, **2f**, **2g**, **2h**, and bortezomib were found to be 1.3, 0.9, 0.5, 1.0, and 1.3%, respectively. Based on the data, compounds **2e** and **2h** induced apoptosis in HL-60 cells more than compounds **2f** and **2g**.

**Table 2:** Percents of Typical Quadrant Analysis of HL-60 Cells Treated with Compounds **2e**, **2f**, **2g**, **2h**, and Bortezomib

	HL-60 cell line					
compound	viable %	necrosis %	early apoptosis %	late apoptosis %	caspase 3(+)	caspase 3(−)
**2e**	56.2	0.0	42.5	1.3	49.1	50.3
**2f**	65.7	0.0	33.4	0.9	62.7	37.0
**2g**	69.7	0.0	29.9	0.5	53.9	45.8
**2h**	54.6	0.0	44.4	1.0	51.3	48.6
**Bortezomib**	23.1	0.0	75.6	1.3	47.0	52.8

**Figure 5:**
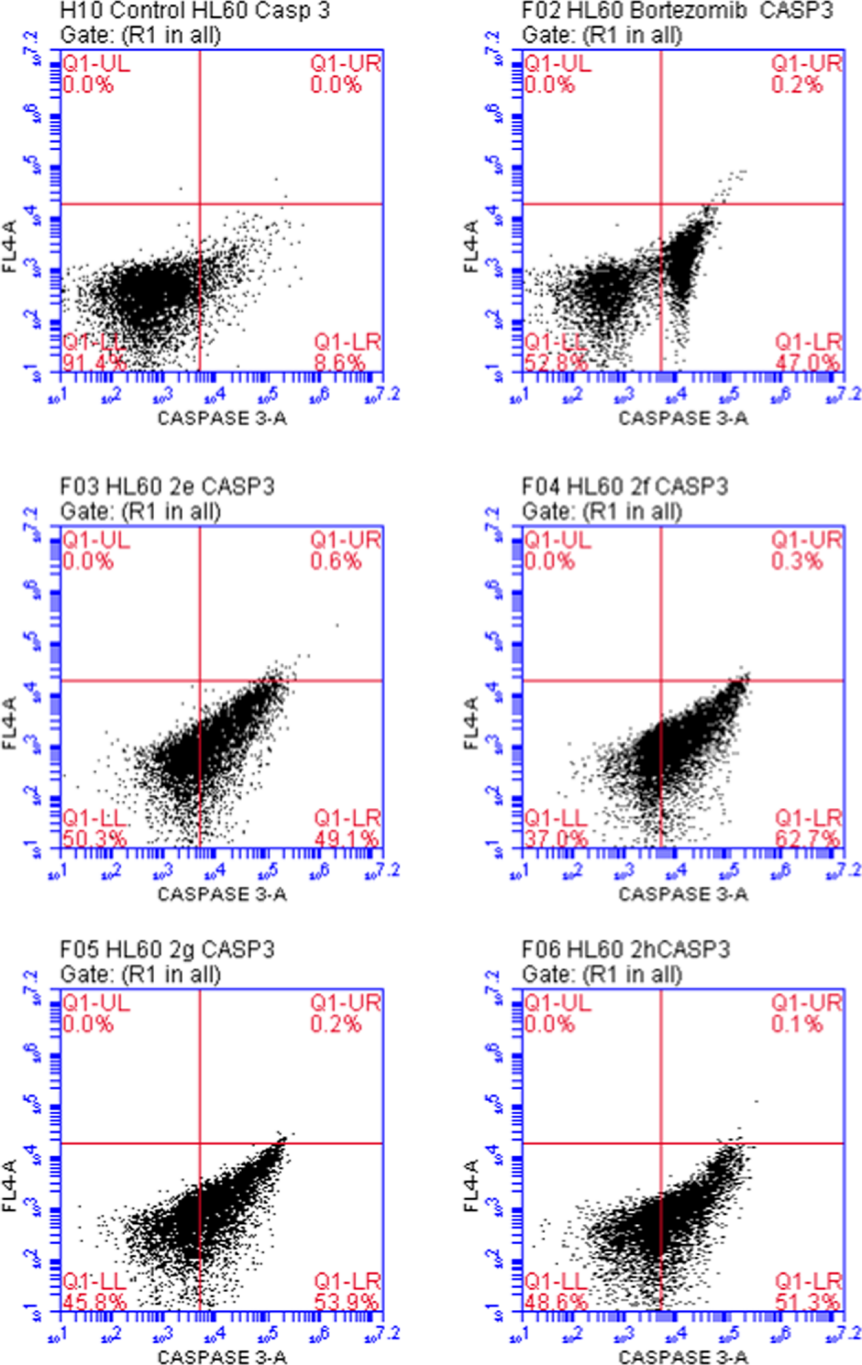
Flow cytometric analysis of caspase 3 activity in HL-60 cells treated with the IC_50_ values of compounds **2e**–**h** and bortezomib.

As depicted in Figure 5, the percentages of caspase 3(+) HL-60 cells treated with compounds **2e**, **2f**, **2g**, **2h**, and bortezomib were found as 49.1, 62.7, 53.9, 51.3, and 47.0%, respectively. According to these findings, compounds **2e**, **2f**, **2g**, and **2h** induced caspase 3 more than bortezomib. It can be concluded that these compounds trigger apoptotic pathway mediated by significant activation of caspase 3 in HL-60 cells.

**Figure 6:**
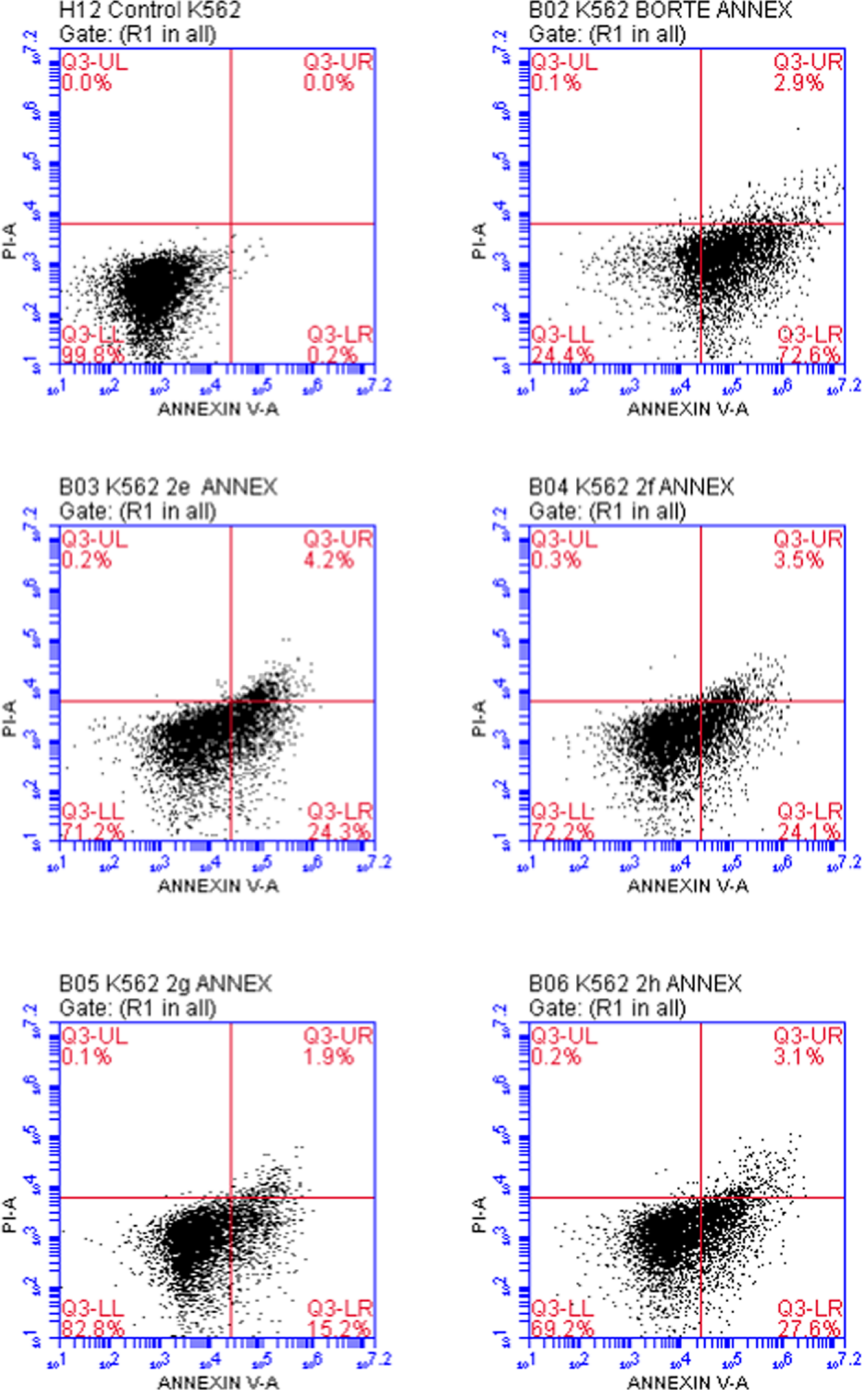
Flow cytometric analysis of K562 cells treated with IC_50_ values of compounds **2e**–**h** and bortezomib.

The percentages of K562 cells undergoing early apoptosis exposed to compounds **2e**, **2f**, **2g**, **2h**, and bortezomib at IC_50_ concentrations were found to be 24.3, 24.1, 15.2, 27.6, and 72.6%, respectively (Table 3, Figure 6). The percentages of K562 cells undergoing late apoptosis exposed to compounds **2e**, **2f**, **2g**, **2h**, and bortezomib were found to be 4.2, 3.5, 1.9, 3.1, and 2.9%, respectively. According to these findings, compounds **2e**, **2f**, and **2h** induced apoptosis in K562 cells more than compound **2g**.

**Table 3:** Percents of Typical Quadrant analysis of K562 Cells Treated with Compounds **2e**, **2f**, **2g**, **2h**, and Bortezomib

	K562 cell line					
compound	viable %	necrosis %	early apoptosis %	late apoptosis %	caspase 3(+)	caspase 3(−)
**2e**	71.2	0.2	24.3	4.2	58.6	41.0
**2f**	72.2	0.3	24.1	3.5	30.1	69.8
**2g**	82.8	0.1	15.2	1.9	10.8	89.2
**2h**	69.2	0.2	27.6	3.1	57.2	42.7
**Bortezomib**	24.4	0.1	72.6	2.9	19.7	80.2

**Figure 7:**
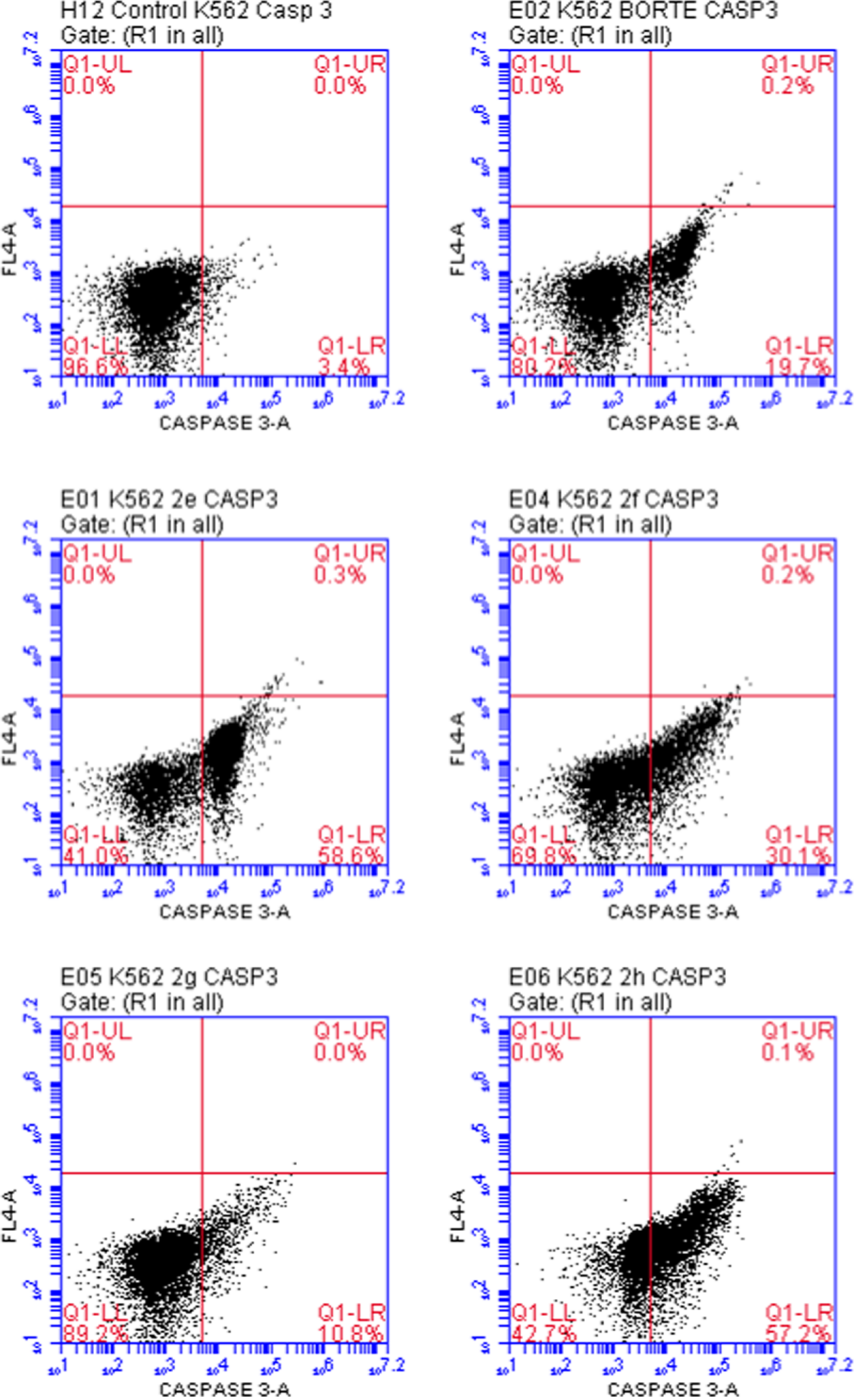
Flow cytometric analysis of caspase 3 activity in K562 cells treated with the IC_50_ values of compounds **2e**–**h** and bortezomib.

As depicted in Figure 7, the percentages of caspase 3(+) K562 cells treated with compounds **2e**, **2f**, **2h**, and bortezomib were found as 58.6, 30.1, 57.2, and 19.7%, respectively. Based on the data, compounds **2e**, **2f**, and **2h** caused caspase 3 induction more than bortezomib. It can be concluded that compounds **2e**, **2f**, and **2h** promote apoptosis in K562 cells via significant caspase 3 activation.

## 3 Conclusions

In this study, the synthesis of 12 new pyrazolines (**2a**–**l**) was performed efficiently. Their cytotoxic features on human leukemia cell lines (HL-60, K562, and THP-1) were determined by means of the WST-1 assay. Compounds **2e**, **2f**, **2g**, and **2h** were determined as promising antileukemic agents on HL-60 and K562 cells. Compound **2f** showed cytotoxic activity against the HL-60 cell line similar to bortezomib. Compound **2h** was more effective on the K562 cell line than bortezomib. To gain further insight into its mode of antileukemic action, *in vitro* mechanistic studies were conducted. Compounds **2f** and **2h** stand out as striking antileukemic agents with marked cytotoxic effects on HL-60 and K562 cells through the induction of apoptosis mediated by caspase 3 activation.

## 4 Materials and Methods

### 4.1 Chemistry

All chemicals procured from commercial suppliers were used without further purification. Melting points (Mp) were determined on an Electrothermal IA9200 melting point apparatus (Staffordshire, UK) and are uncorrected. IR spectra were recorded on an IRPrestige-21 Fourier Transform IR spectrophotometer (Shimadzu, Tokyo, Japan). ^1^H and ^13^C NMR spectra were recorded on an NMR spectrometer (Bruker, Billerica, MA, USA). HRMS spectra were recorded on an LCMS-IT-TOF system (Shimadzu, Kyoto, Japan). Thin-layer chromatography (TLC) was used to track the progress of the chemical reactions and examine the purity of the synthesized compounds.

#### 4.1.1 General Procedure for the Synthesis of 3-(4-(4-Methylpiperazin-1-yl)phenyl)-1-(thiophen-3-yl)prop-2-en-1-one (**1**)

The compound was obtained according to the procedure reported by our research team.^23,27^

Yellow powder. Yield: 60%. Mp 169–170 °C. IR ν_max_ (cm^–1^): 3103.46, 2970.38, 2937.59, 2845.00, 2792.93, 2748.56, 1645.28, 1606.70, 1577.77, 1550.77, 1508.33, 1462.04, 1446.61, 1409.96, 1381.03, 1348.24, 1328.95, 1313.52, 1290.38, 1253.73, 1224.80, 1192.01, 1166.93, 1139.93, 1064.71,1026.13, 1001.06, 981.77, 948.98, 920.05, 885.33, 864.11, 858.32, 802.39, 763.81, 738.74, 684.73, 630.72, 613.36. ^1^H NMR (300 MHz, DMSO-*d*_6_) δ (ppm): 2.22 (s, 3H), 2.44 (t, *J* = 5.04, 5.10, 10.14 Hz, 4H), 2.44 (t, *J* = 5.28, 6.66, 11.94 Hz, 4H), 7.00 (d, *J* = 8.85 Hz, 2H), 7.62 (s, 2H), 7.65 (t, *J* = 1.59, 0.93, 2.52 Hz, 2H), 7.71 (d, *J* = 8.97 Hz, 2H), 8.74–8.75 (m, 1H). ^13^C NMR (75 MHz, DMSO-*d*_6_) δ (ppm): 44.22 (CH_3_), 47.28 (2CH_2_), 54.84 (2CH_2_), 114.72 (2CH), 119.07 (CH), 124.68 (C), 127.62 (CH), 127.91 (CH), 130.94 (2CH), 133.79 (CH), 143.83 (C), 144.01 (CH), 152.92 (C), 182.00 (C). HRMS (ESI) (*m*/*z*) [M + H]^+^ calcd for C_18_H_20_N_2_OS: 313.1369, found: 313.1383.

#### 4.1.2 General Procedure for the Synthesis of 1-Aryl-3-(thiophen-3-yl)-5-(4-(4-methylpiperazin-1-yl)phenyl)-2-pyrazolines (**2a**–**l**)

A mixture of compound **1** (10.0 mmol) and arylhydrazine hydrochloride (20.0 mmol) in the presence of absolute ethanol (35 mL) was refluxed for 22 h. After the experiment was completed by TLC check, the mixture was poured into crushed ice. The precipitate was separated by filtration, washed with water, and crystallized from ethanol.^28^

##### 4.1.2.1 1-Phenyl-3-(thiophen-3-yl)-5-(4-(4-methylpiperazin-1-yl)phenyl)-2-pyrazoline (**2a**)

Brown powder. Yield: 71%. Mp 117–118 °C. IR ν_max_ (cm^–1^): 3105.39, 2960.73, 2839.22, 1610.56, 1595.13, 1514.12, 1496.76, 1454.33, 1394.53, 1359.82, 1336.67, 1292.31, 1244.09, 1188.15, 1159.22, 1134.14, 1089.78, 1051.20, 1026.13, 981.77, 918.12, 873.75, 852.54, 839.03, 821.68, 786.96, 752.24, 692.44, 665.44, 630.72. ^1^H NMR (300 MHz, DMSO-*d*_6_) δ (ppm): 2.73 (s, 3H), 3.01 (dd, *J*_AB_ = 17.43 Hz, *J*_AX_ = 6.18 Hz, 1H), 3.21–3,27 (m, 8H), 3.83 (dd, *J*_BA_ = 17.43 Hz, *J*_BX_ = 12.03 Hz, 1H), 5.35 (dd, *J*_BX_ = 11.94 Hz, *J*_AX_ = 6.09 Hz, 1H), 6.68 (t, *J* = 7.26, 14.52 Hz, 1H), 6.89–6.99 (m, 4H), 7.10–7.17 (m, 4H), 7.63–7.65 (m, 2H), 7.69–7.71 (m, 1H). ^13^C NMR (75 MHz, DMSO-*d*_6_) δ (ppm): 42.81 (CH_2_), 44.30 (CH_3_), 46.16 (2CH_2_), 52.87 (2CH_2_), 62.71 (CH), 113.33 (2CH), 115.61 (CH), 116.77 (2CH), 118.68 (d, *J* = 7.50 Hz, CH), 124.73 (CH), 125.74 (d, *J* = 13.50 Hz, CH), 127.49 (d, *J* = 51.00 Hz, 2CH), 129.48 (d, *J* = 30.00 Hz, 2CH), 130.00 (C), 134.16 (C), 135.27 (C), 144.84 (C), 149.35 (C). HRMS (*m*/*z*): [M + H]^+^ calcd for C_24_H_26_N_4_S: 403.1951. Found: 403.1967.

##### 4.1.2.2 1-(4-Methylsulfonylphenyl)-3-(thiophen-3-yl)-5-(4-(4-methylpiperazin-1-yl)phenyl)-2-pyrazoline (**2b**)

Orange powder. Yield: 53%. Mp 187–188 °C. IR ν_max_ (cm^–1^): 3099.61, 3010.88, 2920.23, 2839.22, 1589.34, 1504.48, 1456.26, 1421.54, 1396.46, 1373.32, 1317.38, 1286.52, 1244.09, 1190.08, 1130.29, 1087.85, 1024.20, 1001.06, 983.70, 954.76, 918,12, 873.75, 825.53, 769.60, 705.95, 634.58, 569.00. ^1^H NMR (300 MHz, DMSO-*d*_6_) δ (ppm): 2.77 (s, 3H), 3.06 (s, 3H), 3.12–3.13 (m, 1H), 3.21–3.27 (m, 8H), 3.92 (dd, *J*_BA_ = 17.79 Hz, *J*_BX_ = 11.94 Hz, 1H), 5.53 (dd, *J*_BX_ = 11.80 Hz, *J*_AX_ = 4.62 Hz, 1H), 6.96 (d, *J* = 8.79 Hz, 2H), 7.09 (d, *J* = 8.94 Hz, 2H), 7.14 (d, *J* = 8.70 Hz, 2H), 7.60–7.62 (m, 2H), 7.65–7.69 (m, 2H), 7.84 (dd, *J* = 1.20, 2.82 Hz, 1H). ^13^C NMR (75 MHz, DMSO-*d*_6_) δ (ppm): 42.55 (CH_2_), 44.36 (CH_3_), 44.62 (CH_3_), 45.88 (2CH_2_), 52.68 (2CH_2_), 61.80 (CH), 112.48 (2CH), 116.89 (2CH), 125.76 (CH), 126.48 (CH), 127.02 (CH), 127.35 (C), 128.15 (2CH), 129.04 (d, *J* = 8.25 Hz, 2CH), 129.60 (d, *J* = 45.00 Hz, C), 133.09 (C), 134.58 (C), 147.77 (d, *J* = 29.25 Hz, C), 149.16 (C). HRMS (*m*/*z*): [M + H]^+^ calcd for C_25_H_28_N_4_O_2_S_2_: 481.1726. Found: 481.1736.

##### 4.1.2.3 1-(4-Methylphenyl)-3-(thiophen-3-yl)-5-(4-(4-methylpiperazin-1-yl)phenyl)-2-pyrazoline (**2c**)

Dark orange powder. Yield: 70%. Mp 132–133 °C. IR ν_max_ (cm^–1^): 3101.54, 3034.33, 2954.95, 2918.30, 2846.93, 1610.56, 1573.91, 1512.19, 1454.33, 1415.75, 1394.53, 1338.60, 1244.09, 1186.22, 1159.22, 1107.14, 1087.85, 1053.13, 1026.13, 981.77, 920.05, 854.47, 819.75, 786.96, 731.02, 688.59, 667.37, 624.94, 617.22, 594.08. ^1^H NMR (300 MHz, DMSO-*d*_6_) δ (ppm): 2.15 (s, 3H), 2.73 (s, 3H), 2.99 (dd, *J*_BA_ = 17.34 Hz, *J*_BX_ = 6.33 Hz, 1H), 3.19–3.26 (m, 8H), 3.80 (dd, *J*_BA_ = 17.31 Hz, *J*_BX_ = 12.12 Hz, 1H), 5.31 (dd, *J*_BX_ = 11.73 Hz, *J*_AX_ = 6.09 Hz, 1H), 6.87 (d, *J* = 8.67 Hz, 2H), 6.94 (d, *J* = 8.28 Hz, 4H), 7.54–7.56 (m, 2H), 7.61–7.68 (m, 2H), 7.72–7.75 (m, 1H). ^13^C NMR (75 MHz, DMSO-*d*_6_) δ (ppm): 20.61 (CH_3_), 44.25 (CH_2_), 44.41 (CH_3_), 46.23 (2CH_2_), 52.91 (2CH_2_), 61.32 (CH), 113.51 (2CH), 116.72 (2CH), 125.69 (CH), 126.33 (CH), 127.28 (d, *J* = 13.50 Hz, 2CH), 127.77 (CH), 128.83 (C), 129.70 (2CH), 134.23 (C), 135.47 (C), 144.34 (C), 145.52 (C), 150.35 (C). HRMS (*m*/*z*): [M + H]^+^ calcd for C_25_H_28_N_4_S: 417.2107. Found: 417.2119.

##### 4.1.2.4 1-(4-Cyanophenyl)-3-(thiophen-3-yl)-5-(4-(4-methylpiperazin-1-yl)phenyl)-2-pyrazoline (**2d**)

Dark golden rod powder. Yield: 85%. Mp 169–170 °C. IR ν_max_ (cm^–1^): 3009.14, 2953.02, 2877.79, 2216.21, 1602.85, 1514.12, 1458.18, 1442.75, 1365.60, 1338.60, 1263.37, 1182.36, 1116.78, 1053.13, 1031.92, 983.70, 916.19, 846.75, 756.10, 638.44, 599.86. ^1^H NMR (300 MHz, DMSO-*d*_6_) δ (ppm): 2.68 (s, 3H), 3.11 (dd, *J*_BA_ = 17.67 Hz, *J*_BX_ = 4.77 Hz, 1H), 3.20–3.26 (m, 8H), 3.91 (dd, *J*_BA_ = 17.79 Hz, *J*_BX_ = 12.00 Hz, 1H), 5.52 (dd, *J*_BX_ = 11.91 Hz, *J*_AX_ = 4.80 Hz, 1H), 6.94 (d, *J* = 8.70 Hz, 2H), 7.04 (d, *J* = 8.88 Hz, 2H), 7.11 (d, *J* = 8.73 Hz, 2H), 7.54 (d, *J* = 8.97 Hz, 2H), 7.59–7.61 (m, 1H), 7.66–7.69 (m, 1H), 7.84–7.85 (m, 1H). ^13^C NMR (75 MHz, DMSO-*d*_6_) δ (ppm): 42.92 (CH_2_), 44.32 (CH_3_), 46.29 (2CH_2_), 53.10 (2CH_2_), 61.76 (CH), 98.93 (C), 113.10 (2CH), 116.77 (2CH), 120.55 (CH), 125.75 (CH), 126.97 (2CH), 127.91 (C), 128.16 (CH), 133.74 (2CH), 134.53 (2C), 147.05 (C), 148.19 (C), 154.58 (C). HRMS (*m*/*z*): [M + H]^+^ calcd for C_25_H_25_N_5_S: 428.1903. Found: 428.1913.

##### 4.1.2.5 1-(4-Bromophenyl)-3-(thiophen-3-yl)-5-(4-(4-methylpiperazin-1-yl)phenyl)-2-pyrazoline (**2e**)

Carrot orange powder. Yield: 80%. Mp 140–141 °C. IR ν_max_ (cm^–1^): 3099.61, 2953.02, 2839.22, 1610.56, 1589.34, 1514.12, 1489.05, 1454.33, 1396.46, 1361.74, 1336.67, 1305.81, 1244.09, 1188.15, 1161.15, 1126.43, 1087.85, 1072.42, 1024.20, 1008.77, 981.77, 918.12, 871.82, 815.89, 783.10, 694.37, 634.58. ^1^H NMR (300 MHz, DMSO-*d*_6_) δ (ppm): 2.76 (s, 3H), 3.04 (dd, *J*_BA_ = 17.46 Hz, *J*_BX_ = 5.70 Hz, 1H), 3.19–3.26 (m, 8H), 3.85 (dd, *J*_BA_ = 17.52 Hz, *J*_BX_ = 11.97 Hz, 1H), 5.38 (dd, *J*_BX_ = 11.76 Hz, *J*_AX_ = 5.55 Hz, 1H), 6.91 (d, *J* = 9.03 Hz, 2H), 6.95 (d, *J* = 8.64 Hz, 2H), 7.13 (d, *J* = 8.73 Hz, 2H), 7.28 (d, *J* = 8.97 Hz, 2H), 7.55–7.57 (m, 1H), 7.63–7.65 (m, 1H), 7.73–7.75 (m, 1H). ^13^C NMR (75 MHz, DMSO-*d*_6_) δ (ppm): 42.72 (CH_2_), 44.40 (CH_3_), 46.07 (2CH_2_), 52.79 (2CH_2_), 62.44 (CH), 109.83 (C), 115.21 (2CH), 116.81 (2CH), 125.47 (d, *J* = 25.50 Hz, CH), 127.13 (2CH), 127.58 (CH), 127.95 (CH), 131.89 (2CH), 133.55 (C), 135.00 (C), 143.88 (C), 145.76 (C), 149.39 (C). HRMS (*m*/*z*): [M + H]^+^ calcd for C_24_H_25_BrN_4_S: 481.1056. Found: 481.1043.

##### 4.1.2.6 1-(4-Fluorophenyl)-3-(thiophen-3-yl)-5-(4-(4-methylpiperazin-1-yl)phenyl)-2-pyrazoline (**2f**)

Brown powder. Yield: 74%. Mp 124–125 °C. IR ν_max_ (cm^–1^): 3014.13, 2972.31, 2864.29, 1558.48, 1541.12, 1508.33, 1458.18, 1363.67, 1338.60, 1290.38, 1182.36, 1118.71, 1064.71, 1031.92, 989.48, 906.54, 655.80. ^1^H NMR (300 MHz, DMSO-*d*_6_) δ (ppm): 2.75 (s, 3H), 3.02 (dd, *J*_BA_ = 17.37 Hz, *J*_BX_ = 6.57 Hz, 1H), 3.19–3.26 (m, 8H), 3.83 (dd, *J*_BA_ = 17.40 Hz, *J*_BX_ = 11.94 Hz, 1H), 5.31 (dd, *J*_BX_ = 11.88 Hz, *J*_AX_ = 6.51 Hz, 1H), 6.90–7.03 (m, 6H), 7.16 (d, *J* = 8.70 Hz, 2H), 7.55–7.57 (m, 1H), 7.63–7.65 (m, 1H), 7.70–7.71 (m, 1H). ^13^C NMR (75 MHz, DMSO-*d*_6_) δ (ppm): 42.70 (CH_2_), 44.47 (CH_3_), 46.06 (2CH_2_), 52.81 (2CH_2_), 63.28 (CH), 114.48 (d, *J* = 7.50 Hz, 2CH), 115.67 (2CH), 115.96 (CH), 116.78 (2CH), 124.86 (C), 125.63 (CH), 127.25 (2CH), 127.86 (CH), 133.89 (C), 135.17 (C), 145.07 (C), 149.37 (C), 157.72 (C). HRMS (*m*/*z*): [M + H]^+^ calcd for C_24_H_25_FN_4_S: 421.1857. Found: 421.1852.

##### 4.1.2.7 1-(4-Chlorophenyl)-3-(thiophen-3-yl)-5-(4-(4-methylpiperazin-1-yl)phenyl)-2-pyrazoline (**2g**)

Tan powder. Yield: 76%. Mp 115–116 °C. IR ν_max_ (cm^–1^): 3003.22, 2953.02, 2879.72, 1612.49, 1597.06, 1516.05, 1494.83, 1456.26, 1363.67, 1338.60, 1246.02, 1184.29, 1116.78, 1091.71, 1055.06, 1031.92, 983.70, 918.12, 842.89, 821.68, 786.96, 615.29, 599.86, 584.43, 567.07, 557.43. ^1^H NMR (300 MHz, DMSO-*d*_6_) δ (ppm): 2.75 (s, 3H), 3.04 (dd, *J*_BA_ = 17.49 Hz, *J*_BX_ = 5.82 Hz, 1H), 3.19–3.26 (m, 8H), 3.85 (dd, *J*_BA_ = 17.49 Hz, *J*_BX_ = 11.97 Hz, 1H), 5.38 (dd, *J*_BX_ = 11.88 Hz, *J*_AX_ = 5.73 Hz, 1H), 6.93–6.97 (m, 4H), 7.12–7.18 (m, 4H), 7.55–7.58 (m, 1H), 7.63–7.66 (m, 1H), 7.73–7.74 (m, 1H). ^13^C NMR (75 MHz, DMSO-*d*_6_) δ (ppm): 42.73 (CH_2_), 44.39 (CH_3_), 46.06 (2CH_2_), 52.80 (2CH_2_), 62.60 (CH), 114.71 (2CH), 116.79 (2CH), 122.21 (CH), 125.22 (C), 125.64 (CH), 127.13 (2CH), 127.94 (C), 129.07 (2CH), 133.59 (CH), 135.01 (C), 143.59 (C), 145.68 (C), 149.41 (C). HRMS (*m*/*z*): [M + H]^+^ calcd for C_24_H_25_ClN_4_S: 437.1561. Found: 437.1561.

##### 4.1.2.8 1-(3-Bromophenyl)-3-(thiophen-3-yl)-5-(4-(4-methylpiperazin-1-yl)phenyl)-2-pyrazoline (**2h**)

Brown powder. Yield: 72%. Mp 122–123 °C. IR ν_max_ (cm^–1^): 3091.89, 2958.80, 2835.36, 1587.42, 1556.55, 1514.12, 1479.40, 1454.33, 1446.61, 1423.47, 1394.53, 1359.82, 1336.67, 1305.81, 1242.16, 1190.08, 1161.15, 1111.00, 1074.35, 1043.49, 1024.20, 997.20, 983.70, 918.12, 871.82, 860.25, 840.96, 819.75, 783.10, 756.10, 711.73, 678.94, 634.58. ^1^H NMR (300 MHz, DMSO-*d*_6_) δ (ppm): 2.80 (s, 3H), 3.05 (dd, *J*_BA_ = 17.46 Hz, *J*_BX_ = 5.52 Hz, 1H), 3.19–3.26 (m, 8H), 3.85 (dd, *J*_BA_ = 17.52 Hz, *J*_BX_ = 11.94 Hz, 1H), 5.41 (dd, *J*_BX_ = 11.94 Hz, *J*_AX_ = 5.37 Hz, 1H), 6.81–6.91 (m, 1H), 6.96 (d, *J* = 8.82 Hz, 2H), 6.98–7.00 (m, 1H), 7.04–7.13 (m, 1H), 7.15 (d, *J* = 8.76 Hz, 2H), 7.18–7.20 (m, 1H), 7.58–7.60 (m, 1H), 7.64–7.66 (m, 1H), 7.76–7.78 (m, 1H). ^13^C NMR (75 MHz, DMSO-*d*_6_) δ (ppm): 42.38 (CH_2_), 44.34 (CH_3_), 45.84 (2CH_2_), 52.60 (2CH_2_), 62.28 (CH), 112.05 (CH), 115.45 (CH), 116.87 (2CH), 120.92 (CH), 122.66 (CH), 125.79 (CH), 127.13 (2CH), 127.96 (C), 130.04 (CH), 131.21 (CH), 133.62 (C), 134.87 (C), 146.00 (C), 146.23 (C), 149.30 (C). HRMS (*m*/*z*): [M + H]^+^ calcd for C_24_H_25_BrN_4_S: 481.1056. Found: 481.1062.

##### 4.1.2.9 1-(3-Chlorophenyl)-3-(thiophen-3-yl)-5-(4-(4-methylpiperazin-1-yl)phenyl)-2-pyrazoline (**2i**)

Brown powder. Yield: 69%. Mp 145–146 °C. IR ν_max_ (cm^–1^): 3008.21, 2972.31, 2868.15, 1593.20, 1516.05, 1456.26, 1363.67, 1244.09, 1184.29, 1118.71, 1058.92, 1031.92, 985.62, 906.54, 854.47, 844.82, 779.24, 680.87, 659.66, 605.65, 565.14. ^1^H NMR (300 MHz, DMSO-*d*_6_) δ (ppm): 2.62 (s, 3H), 3.05 (dd, *J*_BA_ = 17.55 Hz, *J*_BX_ = 5.61 Hz, 1H), 3.19–3.26 (m, 8H), 3.85 (dd, *J*_BA_ = 17.58 Hz, *J*_BX_ = 12.03 Hz, 1H), 5.40 (dd, *J*_BX_ = 11.70 Hz, *J*_AX_ = 5.49 Hz, 1H), 6.68–6.71 (m, 1H), 6.82–6.86 (m, 1H), 6.94 (d, *J* = 8.82 Hz, 2H), 6.96–7.03 (m, 2H), 7.10–7.16 (m, 2H), 7.58–7.60 (m, 1H), 7.64–7.66 (m, 1H), 7.76–7.77 (m, 1H). ^13^C NMR (75 MHz, DMSO-*d*_6_) δ (ppm): 43.53 (CH_2_), 44.36 (CH_3_), 46.58 (2CH_2_), 53.41 (2CH_2_), 62.33 (CH), 111.74 (CH), 112.61 (CH), 115.19 (CH), 116.63 (2CH), 118.02 (CH), 125.77 (CH), 127.07 (2CH), 127.94 (C), 130.92 (CH), 133.22 (CH), 133.97 (C), 134.91 (C), 145.90 (C), 146.19 (C), 149.74 (C). HRMS (*m*/*z*): [M + H]^+^ calcd for C_24_H_25_ClN_4_S: 437.1561. Found: 437.1570.

##### 4.1.2.10 1-(3-Fluorophenyl)-3-(thiophen-3-yl)-5-(4-(4-methylpiperazin-1-yl)phenyl)-2-pyrazoline (**2j**)

Dark brown powder. Yield: 68%. Mp 138–139 °C. IR ν_max_ (cm^–1^): 3078.39, 2956.87, 2837.29, 1608.63, 1575.84, 1514.12, 1490.97, 1454.33, 1396.46, 1363.67, 1338.60, 1307.74, 1271.09, 1244.09, 1178.51, 1151.50, 1112.93, 1091.71, 1066.64, 1056.99, 1026.13, 1006.84, 981.77, 920.05, 869.90, 819.75, 781.17, 761.88, 680.87, 663.51, 634.58, 603.72. ^1^H NMR (300 MHz, DMSO-*d*_6_) δ (ppm): 2.65 (s, 3H), 3.05 (dd, *J*_BA_ = 17.34 Hz, *J*_BX_ = 5.61 Hz, 1H), 3.27 (bs, 4H), 3.40 (bs, 4H), 3.85 (dd, *J*_BA_ = 17.55 Hz, *J*_BX_ = 12.00 Hz, 1H), 5.38 (dd, *J*_BX_ = 11.85 Hz, *J*_AX_ = 5.64 Hz, 1H), 6.43–6.50 (m, 1H), 6.71–6.78 (m, 2H), 6.94 (d, *J* = 8.79 Hz, 2H), 7.13–7.18 (m, 3H), 7.59 (dd, *J* = 1.05 Hz, 5.10 Hz, 1H), 7.65 (dd, *J* = 2.88, 5.04 Hz, 1H), 7.75–7.76 (m, 1H). ^13^C NMR (75 MHz, DMSO-*d*_6_) δ (ppm): 43.24 (CH_2_), 44.38 (CH_3_), 46.35 (2CH_2_), 53.09 (2CH_2_), 62.50 (CH), 99.82 (CH), 100.18 (CH), 104.79 (d, *J* = 21.00 Hz, CH), 109.22 (CH), 116.69 (2CH), 125.62 (d, *J* = 19.50 Hz, CH), 127.09 (2CH), 127.93 (CH), 130.89 (d, *J* = 11.25 Hz, CH), 133.45 (C), 134.94 (C), 145.99 (C), 146.42 (d, *J* = 11.25 Hz, C), 149.64 (C), 163.36 (d, *J* = 238.5 Hz, C). HRMS (*m*/*z*): [M + H]^+^ calcd for C_24_H_25_FN_4_S: 421.1857. Found: 421.1868.

##### 4.1.2.11 1-(3-Nitrophenyl)-3-(thiophen-3-yl)-5-(4-(4-methylpiperazin-1-yl)phenyl)-2-pyrazoline (**2k**)

Dark red powder. Yield: 84%. Mp 120–121 °C. IR ν_max_ (cm^–1^): 3097.68, 2839.22, 1612.49, 1568.13, 1517.98, 1487.12, 1454.33, 1394.53, 1342.46, 1307.74, 1242.16, 1188.15, 1161.15, 1112.93, 1091.71, 1074.35, 1026.13, 1004.91, 983.70, 920.05, 889.18, 871.82, 840.96, 821.68, 785.03, 732.95, 669.30, 651.94, 634.58. ^1^H NMR (300 MHz, DMSO-*d*_6_) δ (ppm): 2.93 (s, 3H), 3.13 (dd, *J*_BA_ = 17.67 Hz, *J*_BX_ = 5.49 Hz, 1H), 3.26 (bs, 4H), 3.40 (bs, 4H), 3.91 (dd, *J*_BA_ = 17.61 Hz, *J*_BX_ = 11.91 Hz, 1H), 5.50 (dd, *J*_BX_ = 11.85 Hz, *J*_AX_ = 5.43 Hz, 1H), 6.94 (d, *J* = 8.76 Hz, 2H), 7.15 (d, *J* = 8.70 Hz, 2H), 7.26–7.30 (m, 1H), 7.38–7.43 (m, 1H), 7.49–7.51 (m, 1H), 7.60–7.69 (m, 2H), 7.77–7.78 (m, 1H), 7.83–7.84 (m, 1H). ^13^C NMR (75 MHz, DMSO-*d*_6_) δ (ppm): 43.84 (CH_2_), 44.55 (CH_3_), 46.75 (2CH_2_), 53.62 (2CH_2_), 62.48 (CH), 106.99 (CH), 112.72 (CH), 115.16 (2CH), 116.57 (CH), 119.11 (CH), 125.76 (CH), 127.14 (2CH), 128.09 (CH), 130.63 (CH), 133.87 (C), 134.64 (C), 145.39 (C), 147.25 (2C), 149.00 (C). HRMS (*m*/*z*): [M + H]^+^ calcd for C_24_H_25_N_5_O_2_S: 448.1802. Found: 448.1810.

##### 4.1.2.12 1-(4-Methoxyphenyl)-3-(thiophen-3-yl)-5-(4-(4-methylpiperazin-1-yl)phenyl)-2-pyrazoline (**2l**)

Dark brown powder. Yield: 73%. Mp 149–150 °C. IR ν_max_ (cm^–1^): 3011.68, 2974.23, 2870.08, 1460.11, 1363.67, 1338.60, 1182.36, 1120.64, 1056.99, 1028.06, 1006.84, 906.54, 821.68, 759.95, 657.73, 626.87, 611.43, 576.72. ^1^H NMR (300 MHz, DMSO-*d*_6_) δ (ppm): 2.76 (s, 3H), 2.95 (dd, *J*_BA_ = 17.40 Hz, *J*_BX_ = 6.90 Hz, 1H), 3.22 (bs, 8H), 3.61–3.71 (bs, 3H), 3.72 (bs, 1H), 5.22 (dd, *J*_BX_ = 11.40 Hz, *J*_AX_ = 6.90 Hz, 1H), 6.73 (d, *J* = 9.00 Hz, 2H), 6.88–6.95 (m, 4H), 7.15 (d, *J* = 8.70 Hz, 2H), 7.52–7.58 (m, 1H), 7.60–7.62 (m, 1H), 7.73–7.76 (m, 1H). ^13^C NMR (75 MHz, DMSO-*d*_6_) δ (ppm): 42.81 (CH_2_), 45.46 (CH_3_), 46.12 (CH_2_), 52.76 (CH_2_), 52.86 (CH_2_), 52.97 (CH_2_), 55.65 (CH_3_), 63.76 (CH), 114.69 (d, *J* = 6.75 Hz, CH), 114.89 (2CH), 115.70 (CH), 116.77 (CH), 124.17 (CH), 125.57 (CH), 127.37 (d, *J* = 10.50 Hz, CH), 127.77 (2CH), 129.61 (CH), 134.36 (C), 135.31 (C), 139.43 (C), 144.19 (C), 149.18 (C), 152.64 (C). HRMS (*m*/*z*): [M + H]^+^ calcd for C_25_H_28_N_4_OS: 433.2057. Found: 433.2039.

### 4.2 Anticancer Activity

#### 4.2.1 Cell Culture

Compounds **2a**–**l** were subjected to the WST-1 assay to determine their cytotoxic features on human leukemia cell lines. The compounds were dissolved in dimethyl sulfoxide (DMSO) at the concentrations of 31.25, 62.5, 125, 250, and 500 μM. HL-60 APL (ATCC CCL-240), K562 CML (ATCC CCL-243), and THP-1 acute monocytic leukemia (ATCC TIB-202) cells were grown in Dulbecco’s modified Eagle’s medium (DMEM) with heat in activated 10% fetal bovine serum (FBS), 100 mg/mL penicillin, 100 mg/mL streptomycin, and 1% l-glutamine. Cells were grown in a humidified atmosphere of 95% air/5% CO_2_ at 37 °C. Bortezomib was used as a positive control.

#### 4.2.2 Determination of Cytotoxicity by the WST-1 Method

WST-1 (4-[3-(4-iodophenyl)-2-(4-nitrophenyl)-2*H*-5-tetrazolio]-1,3-benzene disulfonate) is a tetrazolium salt that specifically binds to the succinate dehydrogenase in the mitochondria of living cells and converts to water-insoluble formazan salts. The absorbance value measured spectrophotometrically in the WST-1 method indicates the metabolic activities of the cells in culture, and this value is related to the number of living cells. As the proliferation increases, the absorbance increases with the formation of formazan salt.^29^ K562, HL-60, and THP-1 cells were treated through decreasing concentrations (500, 250, 125, 62.50, and 31.25 μM) after 24 h of incubation with an equal number of 5 × 10^3^ cells.^30^ The cells were left to incubate for 24 h. At the end of the 24 h incubation period, 20 μL of WST-1 reagent was added to the cells in each well, and the cells were incubated for 3 h in the incubator. At the end of the incubation period, absorbance values at 420 nm, determined using the Cytation 3 Cell Imaging Multi-Mode Reader (BioTek, Santa Clara, USA), were read as 7 replicates (7 wells, 1 blind) for each concentration.

#### 4.2.3 Annexin V–PI Apoptosis Assay by Flow Cytometry

PI and Annexin V are used to detect the viability of cells from differences in the integrity and permeability of the plasma membranes of apoptotic and necrotic cells. PI is used more often than other core dyes due to its stability and its capacity of being a good indicator for cell viability. The release of phosphatidylserine from apoptotic cells inside the healthy cell membranes via disintegration of the cell membrane can be observed with Annexin V–PI to show late stages of cell death, or necrotic cells.^31^ To carry out this study, the protocol of the Annexin V FITC Apoptosis Detection Kit (BD, catalogue no: 556547, San Jose, USA) was applied. K562, HL-60, and THP-1 cells were seeded in medium with six-well plates (1 × 10^5^ cells in each well). IC_50_ values obtained as a result of the WST-1 experiment were applied to the cells. The plates were then incubated for 24 h. At the end of the incubation period, the cells in each of the 6 wells were removed and centrifuged at 1200 rpm for 5 min. The supernatant was then removed, and the kit instructions were applied. Samples were analyzed on a flow cytometer (Becton-Dickinson Accuri C6, Piscataway, USA).

#### 4.2.4 Caspase 3 Activity by Flow Cytometry

Changes in the caspase 3 activity of the cells were examined by PE Active Caspase 3 Apoptosis Kit (BD Pharmingen, cat. no. 550914, San Jose). Caspase 3 is a key protease that is activated during the early stages of apoptosis and, like other members of the caspase family, is synthesized as an inactive proenzyme that is processed in cells undergoing apoptosis by self-proteolysis or cleavage by another protease. In short, the K562, HL-60, and THP-1 cells (1 × 10^5^ cells/well) were seeded in six-well plates and treated with IC_50_ values of compounds **2e**–**h** for 24 h. After the incubations, the analysis was performed according to the kit procedure, processed for data acquisition, and analyzed on a Becton-Dickinson Accuri C6 flow cytometer using Accuri C6 software. At least 10 000 cells were analyzed per sample.

## Supporting Information Available

IR, ^1^H NMR, ^13^C NMR, and HRMS spectra of the most active antileukemic compounds (**2e**, **2f**, **2g**, and **2h**) (PDF)
