# Supplementary material for: Design, Synthesis, and Evaluation of a New Series of 2-Pyrazolines as Potential Antileukemic Agents
Source: ACS Omega. 2023 Oct 30;8(45):42867–77. doi: 10.1021/acsomega.3c05860 (PMC10652261; doi:10.1021/acsomega.3c05860)
Supplement: Supplementary file 1 — ao3c05860_si_001.pdf [file ao3c05860_si_001.pdf]

Supporting Information

Design, Synthesis and Evaluation of a New Series of 2-Pyrazolines as Potential Antileukemic Agents

Mehlika Dilek Altıntop [1], Zerrin Cantürk [2], Ahmet Özdemir [1,*]

[1] *Department of Pharmaceutical Chemistry, Faculty of Pharmacy, Anadolu University, 26470 Eskişehir, Turkey*

[2] *Department of Pharmaceutical Microbiology, Faculty of Pharmacy, Anadolu University, 26470 Eskişehir, Turkey*

* Corresponding author.

List of Contents

**Figure**	**Page**
**Figure S1.** IR spectrum of compound **2e**	**S3**
**Figure S2.** ^{1}H NMR spectrum of compound **2e**	**S4**
**Figure S3.** ^{13}C NMR spectrum of compound **2e**	**S5**
**Figure S4.** HRMS spectrum of compound **2e**	**S6**
**Figure S5.** IR spectrum of compound **2f**	**S7**
**Figure S6.** ^{1}H NMR spectrum of compound **2f**	**S8**
**Figure S7.** ^{13}C NMR spectrum of compound **2f**	**S9**
**Figure S8.** HRMS spectrum of compound **2f**	**S10**
**Figure S9.** IR spectrum of compound **2g**	**S11**
**Figure S10.** ^{1}H NMR spectrum of compound **2g**	**S12**
**Figure S11.** ^{13}C NMR spectrum of compound **2g**	**S13**
**Figure S12.** HRMS spectrum of compound **2g**	**S14**
**Figure S13.** IR spectrum of compound **2h**	**S15**
**Figure S14.** ^{1}H NMR spectrum of compound **2h**	**S16**
**Figure S15.** ^{13}C NMR spectrum of compound **2h**	**S17**
**Figure S16.** HRMS spectrum of compound **2h**	**S18**

Figure S1. IR spectrum of compound **2e**

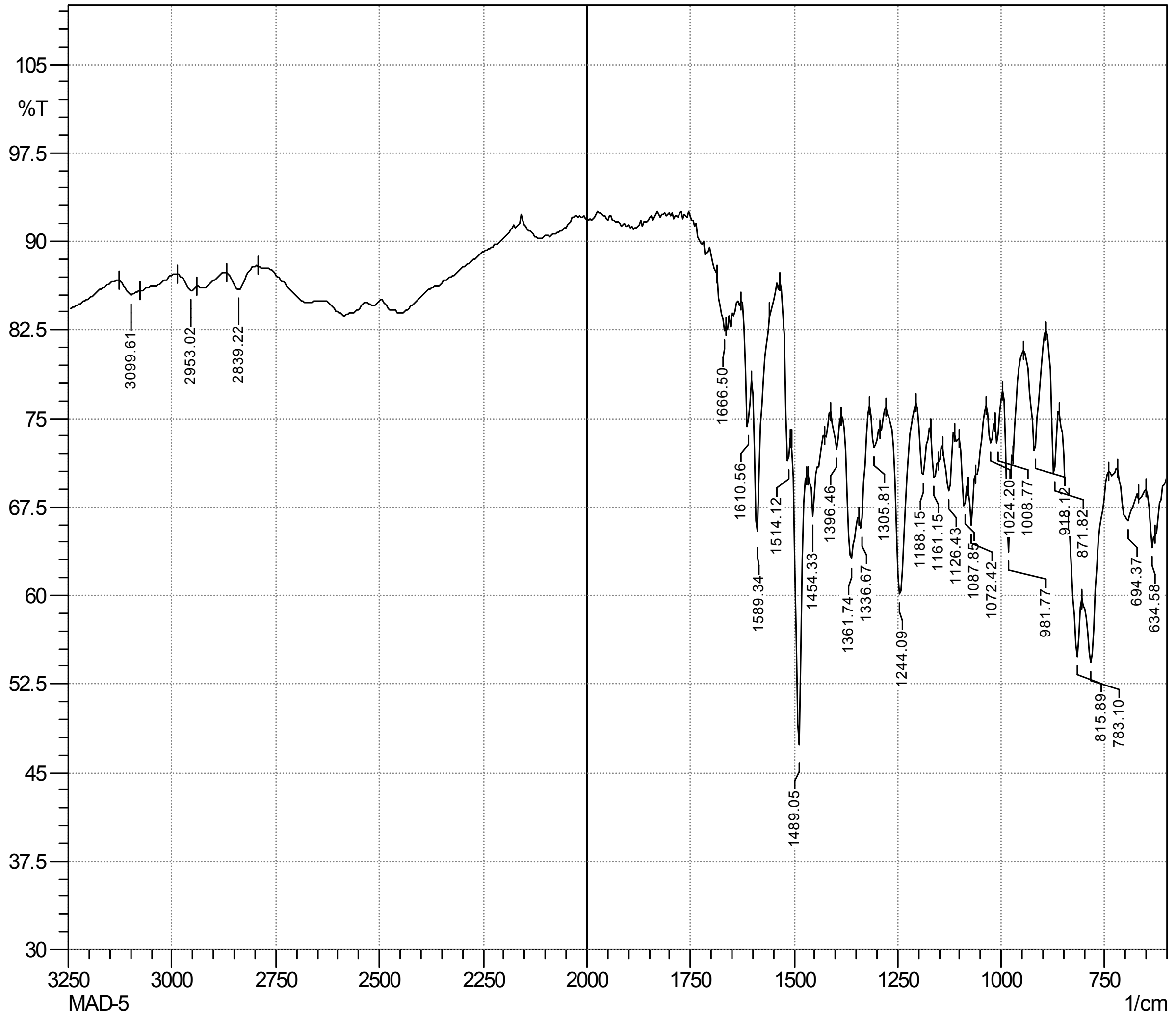

Figure S2. ^{1}H NMR spectrum of compound **2e**

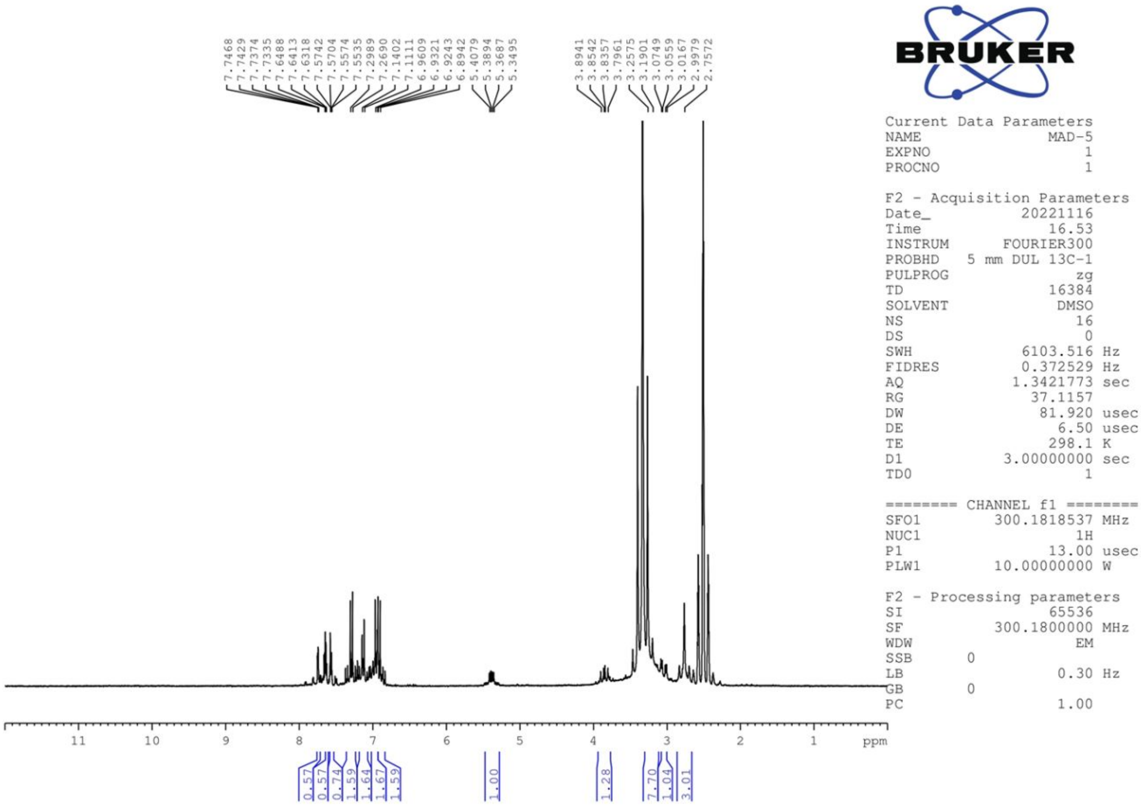

Figure S3. ^{13}C NMR spectrum of compound **2e**

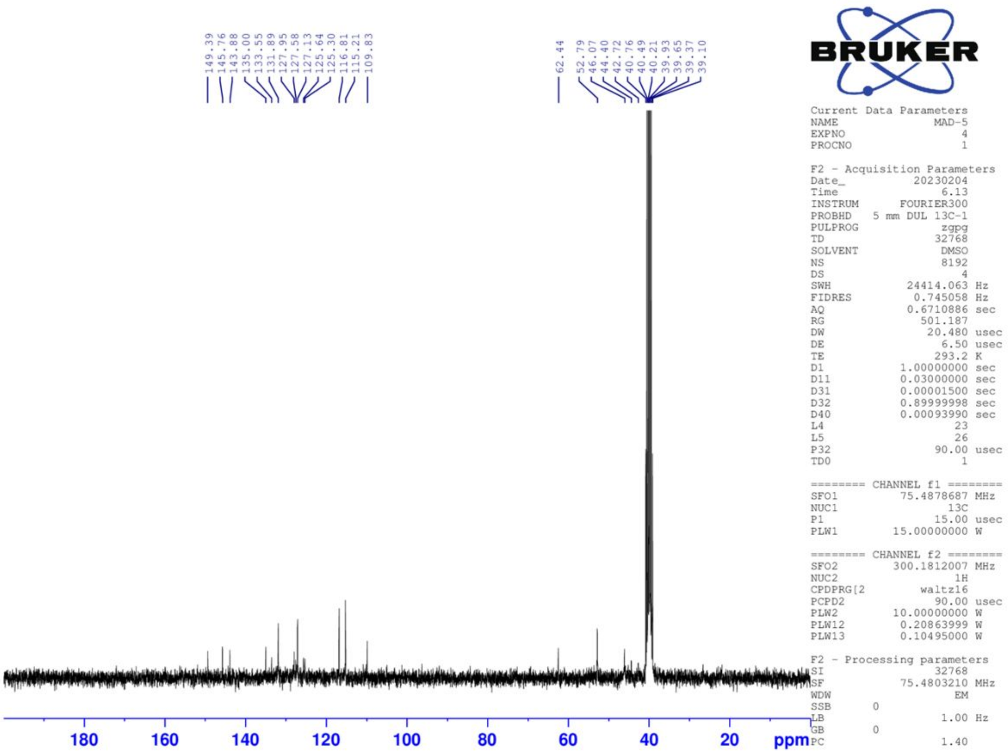

Figure S4. HRMS spectrum of compound **2e**

Formula Predictor Report - MAD-5_1.lcd

Page 1 of 1

Data File: C:\LabSolutions\Data\Analiz\AOzdemir\MAD-5_1.lcd

Elmt	Val.	Min	Max	Elmt	Val.	Min	Max	Elmt	Val.	Min	Max	Elmt	Val.	Min	Max	Use Adduct
H	1	20	30	O	2	0	1	S	2	1	1	Ru	2	0	0	H
C	4	20	30	F	1	0	1	Cl	1	0	1	Pd	2	0	0	
N	3	0	5	P	3	0	0	Br	1	0	1	I	3	0	0	

Error Margin (ppm):	5	DBE Range:	5.0 - 25.0	Electron Ions:	odd
HC Ratio:	unlimited	Apply N Rule:	yes	Use MSn Info:	yes
Max Isotopes:	3	Isotope RI (%):	1.00	Isotope Res:	9000
MSn Iso RI (%):	10.00	MSn Logic Mode:	AND	Max Results:	50

Event#: 1 MS(E+) Ret. Time : 3.560 -> 3.573 Scan# : 535 -> 537

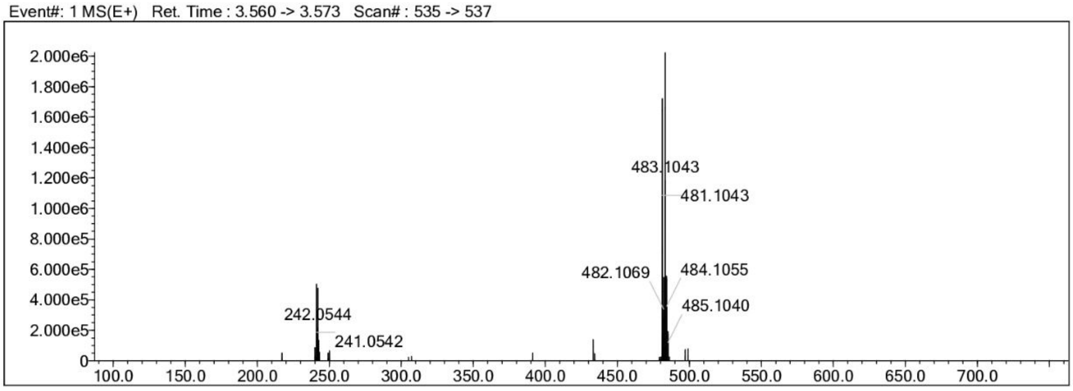

Measured region for 481.1043 m/z

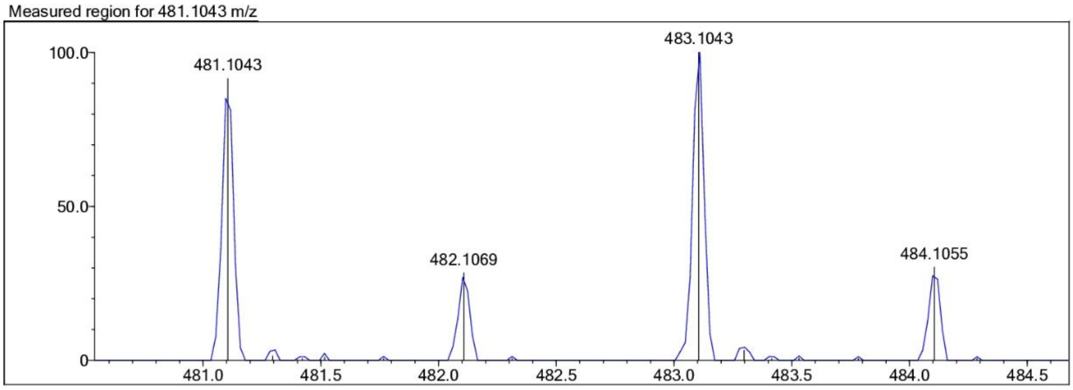

C24 H25 N4 S Br [M+H]+ : Predicted region for 481.1056 m/z

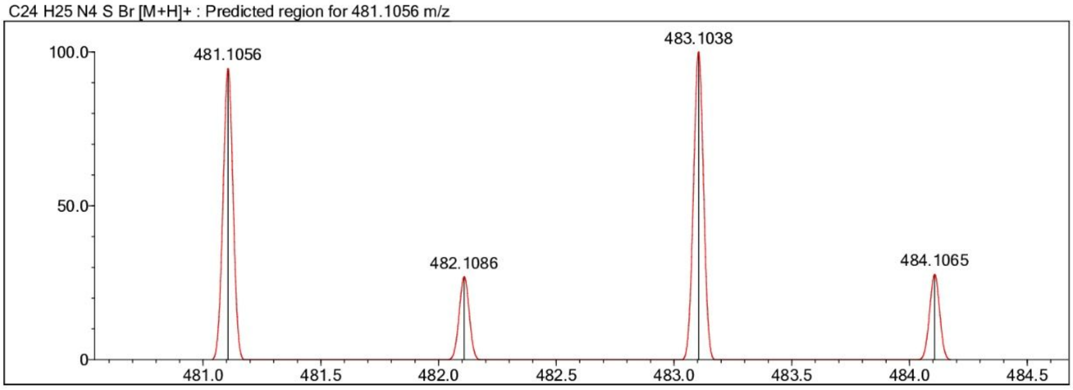

Rank	Score	Formula (M)	Ion	Meas. m/z	Pred. m/z	Df. (mDa)	Df. (ppm)	Iso	DBE
1	72.55	C24 H25 N4 S Br	[M+H]+	481.1043	481.1056	-1.3	-2.70	75.77	14.0

Figure S5. IR spectrum of compound **2f**

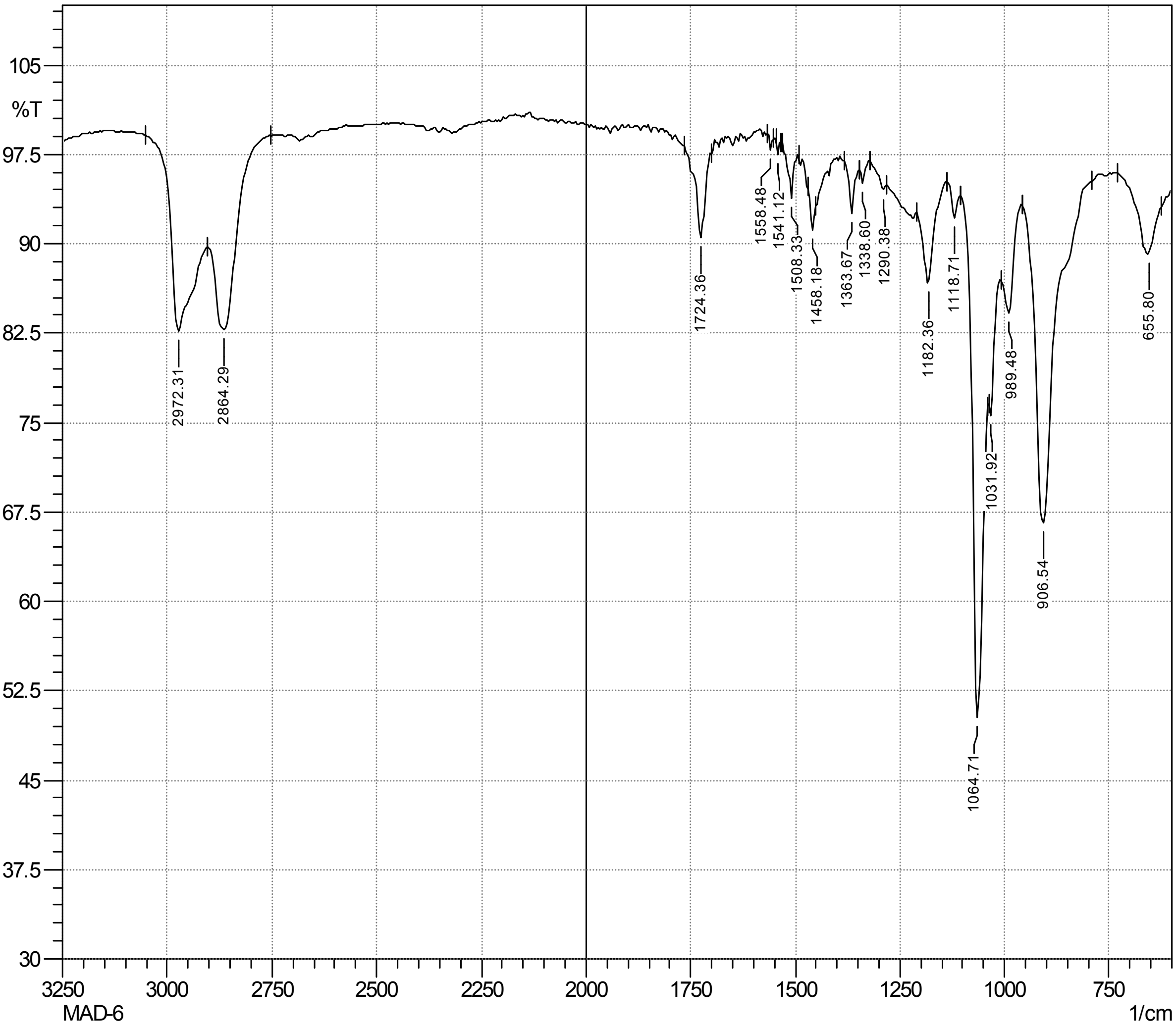

Figure S6. ^{1}H NMR spectrum of compound **2f**

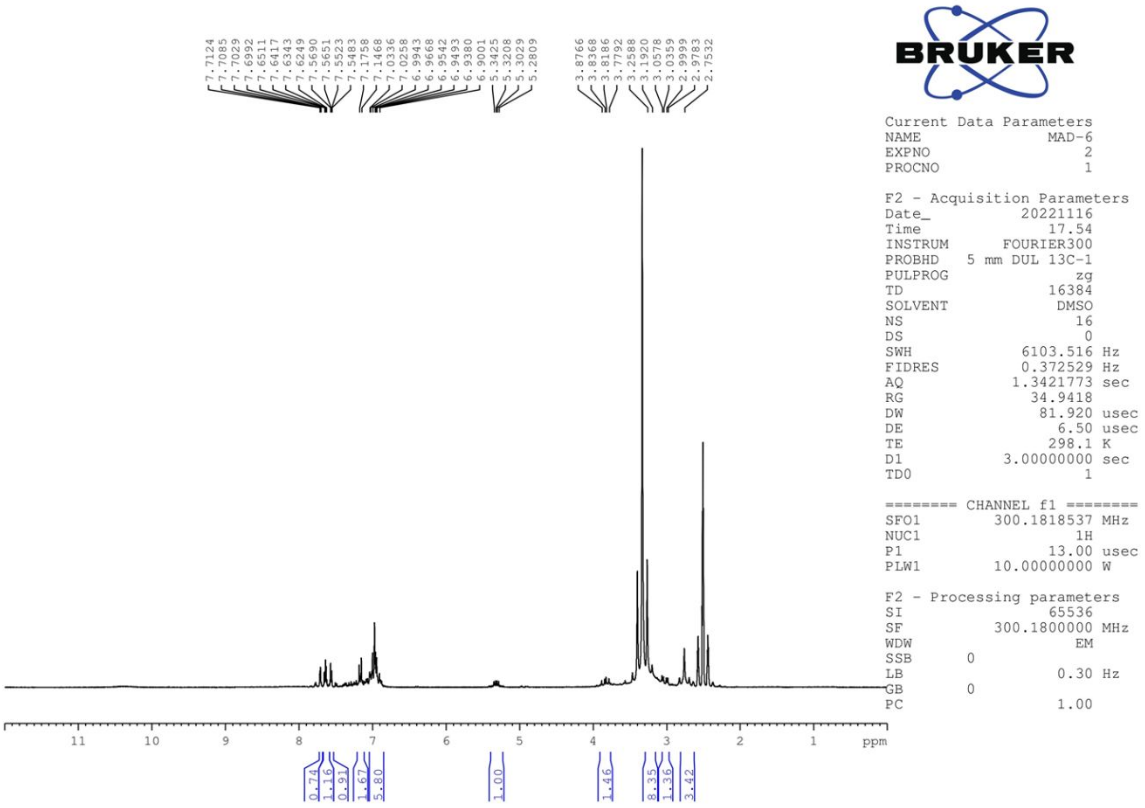

Figure S7. ^{13}C NMR spectrum of compound **2f**

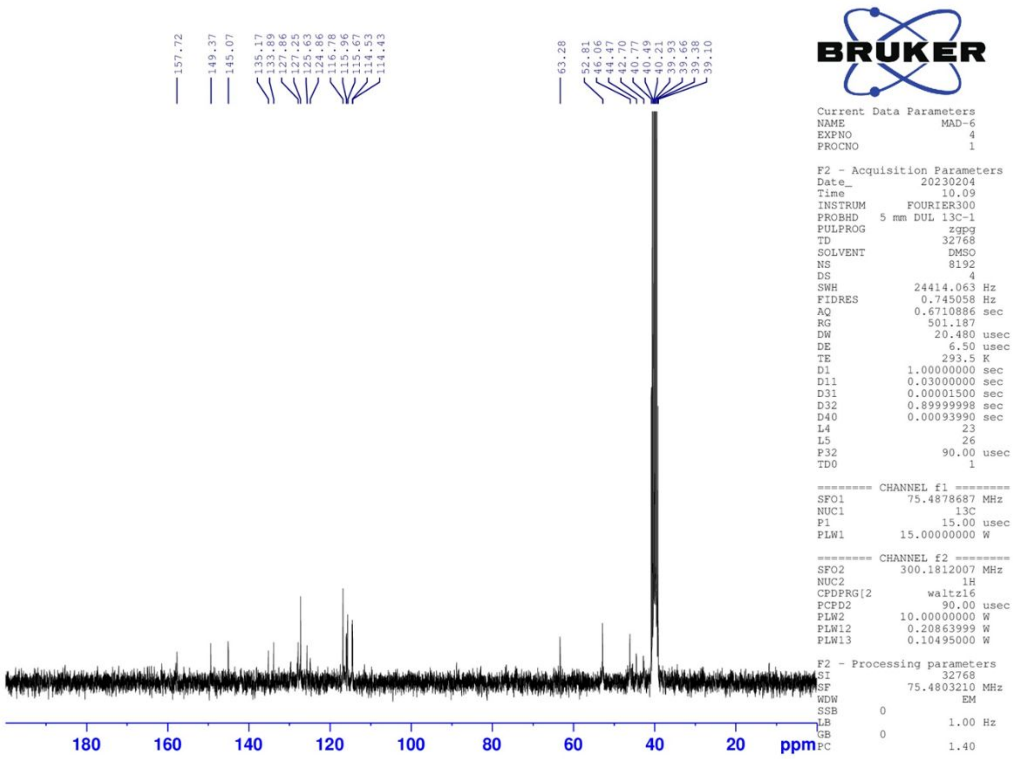

Figure S8. HRMS spectrum of compound **2f**

Data File: C:\LabSolutions\Data\Analiz\AOzdemir\MAD-6_2.lcd

Elmt	Val.	Min	Max	Elmt	Val.	Min	Max	Elmt	Val.	Min	Max	Elmt	Val.	Min	Max	Use Adduct
H	1	20	30	O	2	0	1	S	2	1	1	Ru	2	0	0	H
C	4	20	30	F	1	0	1	Cl	1	0	1	Pd	2	0	0	
N	3	0	5	P	3	0	0	Br	1	0	1	I	3	0	0	

Error Margin (ppm):	5	DBE Range:	5.0 - 25.0	Electron Ions:	odd
HC Ratio:	unlimited	Apply N Rule:	yes	Use MSn Info:	yes
Max Isotopes:	3	Isotope RI (%):	1.00	Isotope Res:	9000
MSn Iso RI (%):	10.00	MSn Logic Mode:	AND	Max Results:	50

Event#: 1 MS(E+) Ret. Time : 3.200 -> 3.200 Scan# : 481 -> 481

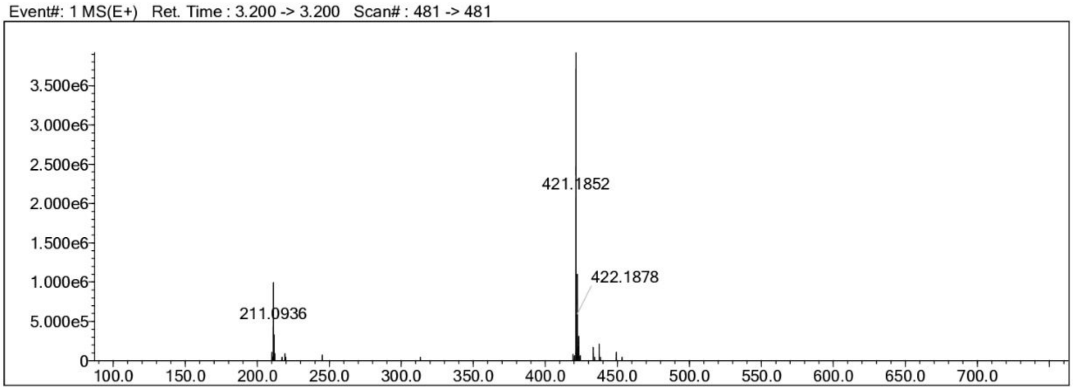

Measured region for 421.1852 m/z

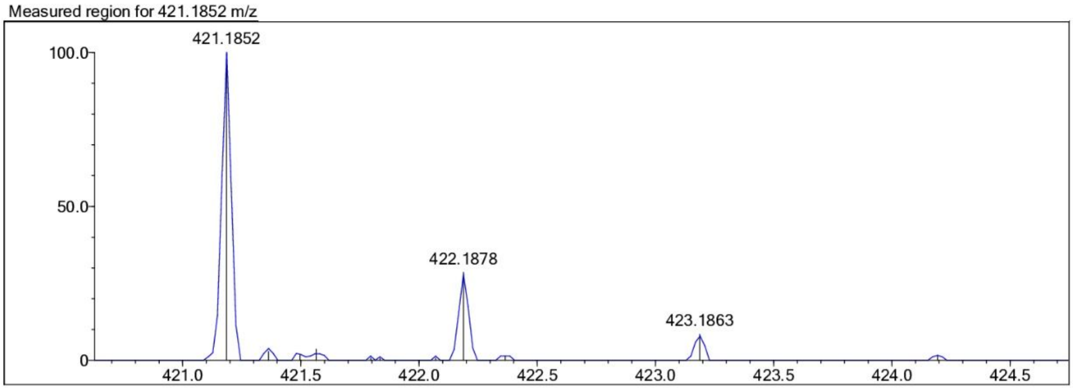

C24 H25 N4 F S [M+H]+ : Predicted region for 421.1857 m/z

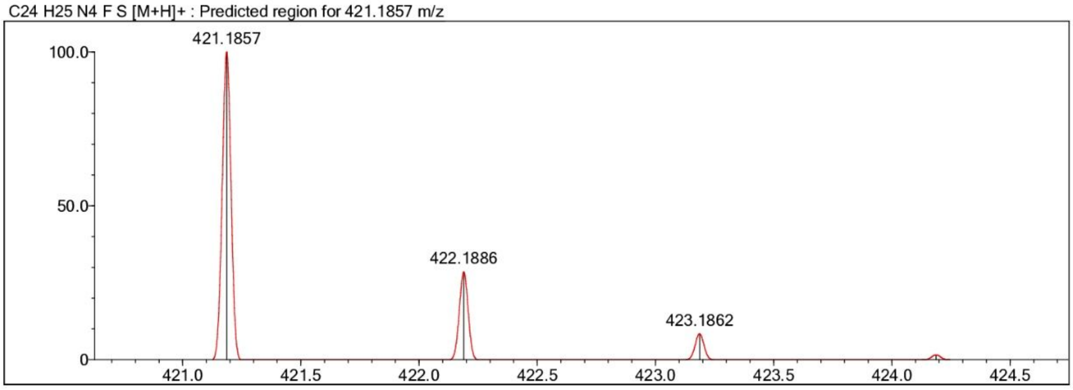

Rank	Score	Formula (M)	Ion	Meas. m/z	Pred. m/z	Df. (mDa)	Df. (ppm)	Iso	DBE
1	92.66	C24 H25 N4 F S	[M+H]+	421.1852	421.1857	-0.5	-1.19	93.10	14.0

Figure S9. IR spectrum of compound **2g**

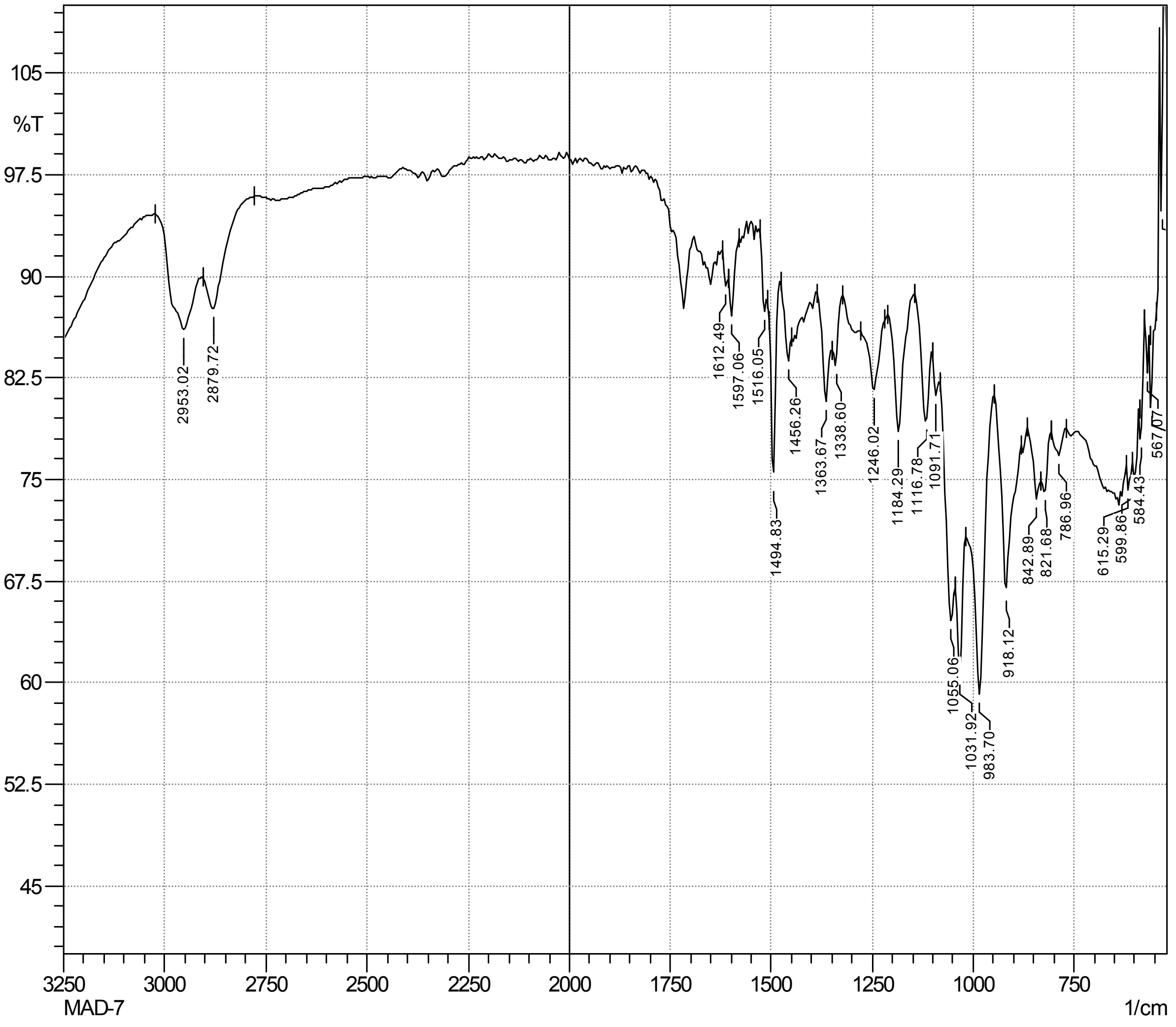

Figure S10. ^{1}H NMR spectrum of compound **2g**

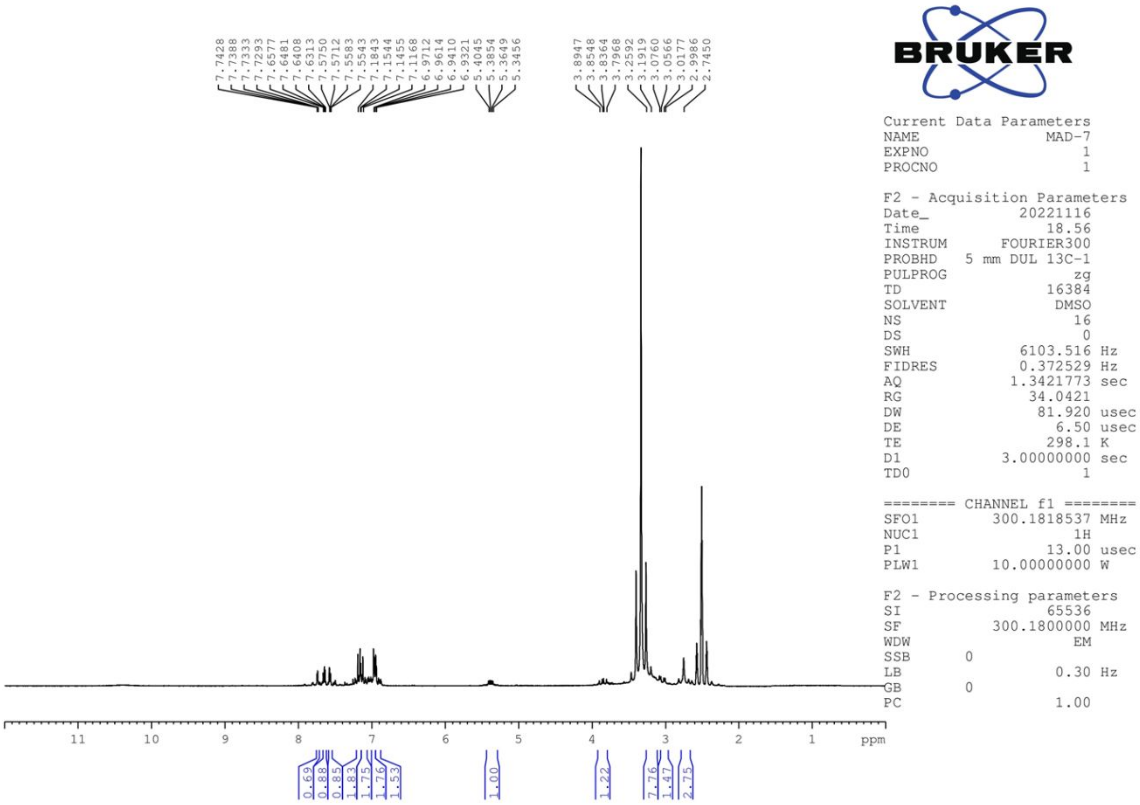

Figure S11. ^{13}C NMR spectrum of compound **2g**

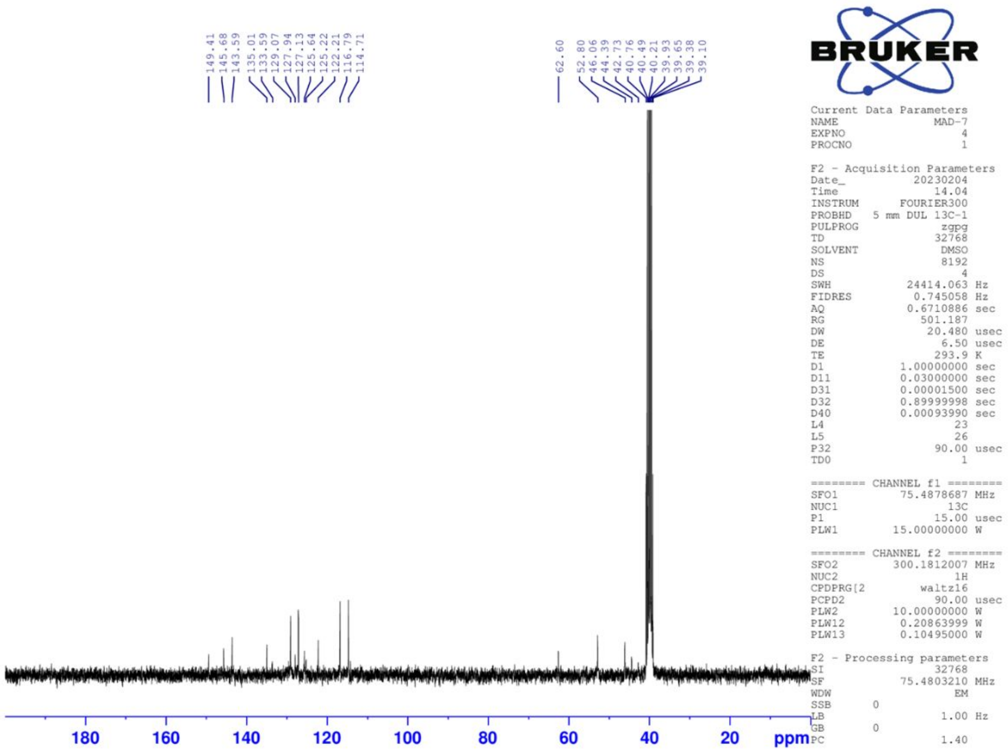

Figure S12. HRMS spectrum of compound **2g**

Data File: C:\LabSolutions\Data\Analiz\AOzdemir\MAD-7_3.lcd

Elmt	Val.	Min	Max	Elmt	Val.	Min	Max	Elmt	Val.	Min	Max	Elmt	Val.	Min	Max	Use Adduct
H	1	20	30	O	2	0	1	S	2	1	1	Ru	2	0	0	H
C	4	20	30	F	1	0	1	Cl	1	0	1	Pd	2	0	0	
N	3	0	5	P	3	0	0	Br	1	0	1	I	3	0	0	

Error Margin (ppm):	5	DBE Range:	11.0 - 25.0	Electron Ions:	odd
HC Ratio:	unlimited	Apply N Rule:	yes	Use MSn Info:	yes
Max Isotopes:	3	Isotope RI (%):	1.00	Isotope Res:	9000
MSn Iso RI (%):	10.00	MSn Logic Mode:	AND	Max Results:	50

Event#: 1 MS(E+) Ret. Time : 3.520 -> 3.520 Scan# : 529 -> 529

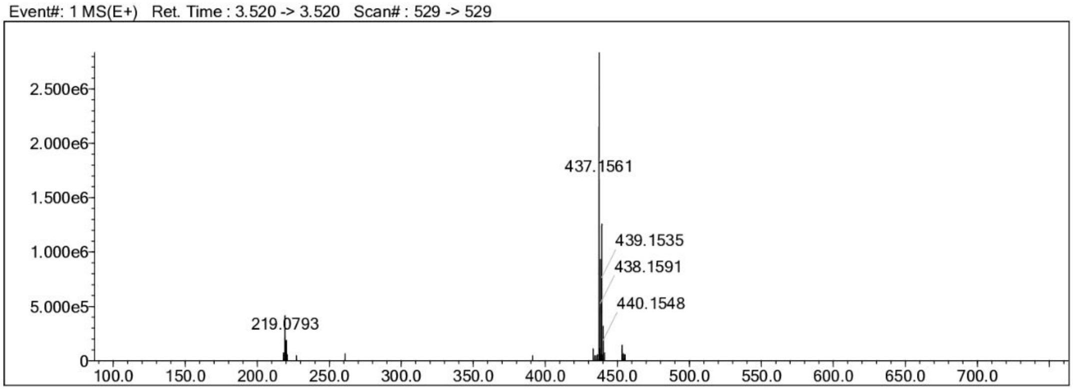

Measured region for 437.1561 m/z

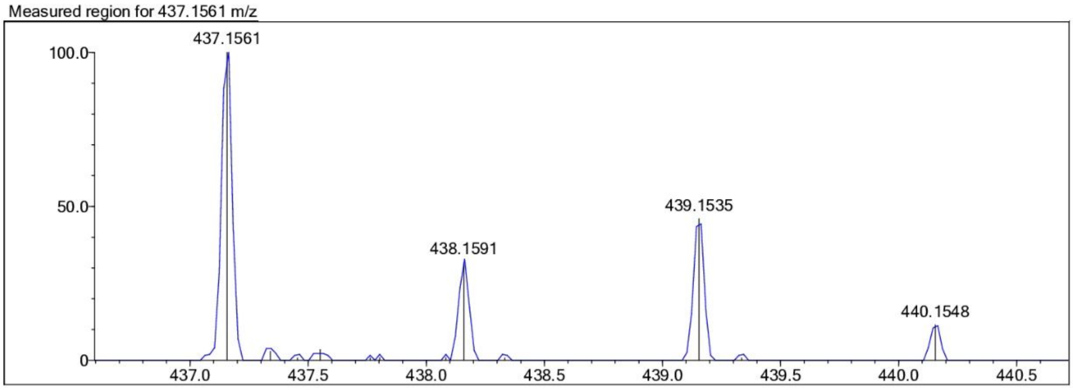

C24 H25 N4 S Cl [M+H]+ : Predicted region for 437.1561 m/z

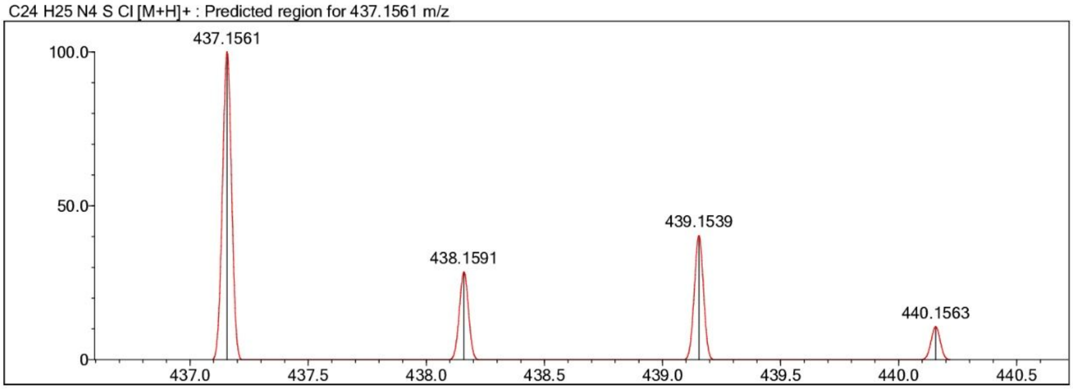

Rank	Score	Formula (M)	Ion	Meas. m/z	Pred. m/z	Df. (mDa)	Df. (ppm)	Iso	DBE
1	92.62	C24 H25 N4 S Cl	[M+H]+	437.1561	437.1561	-0.0	0.00	92.62	14.0

Figure S13. IR spectrum of compound **2h**

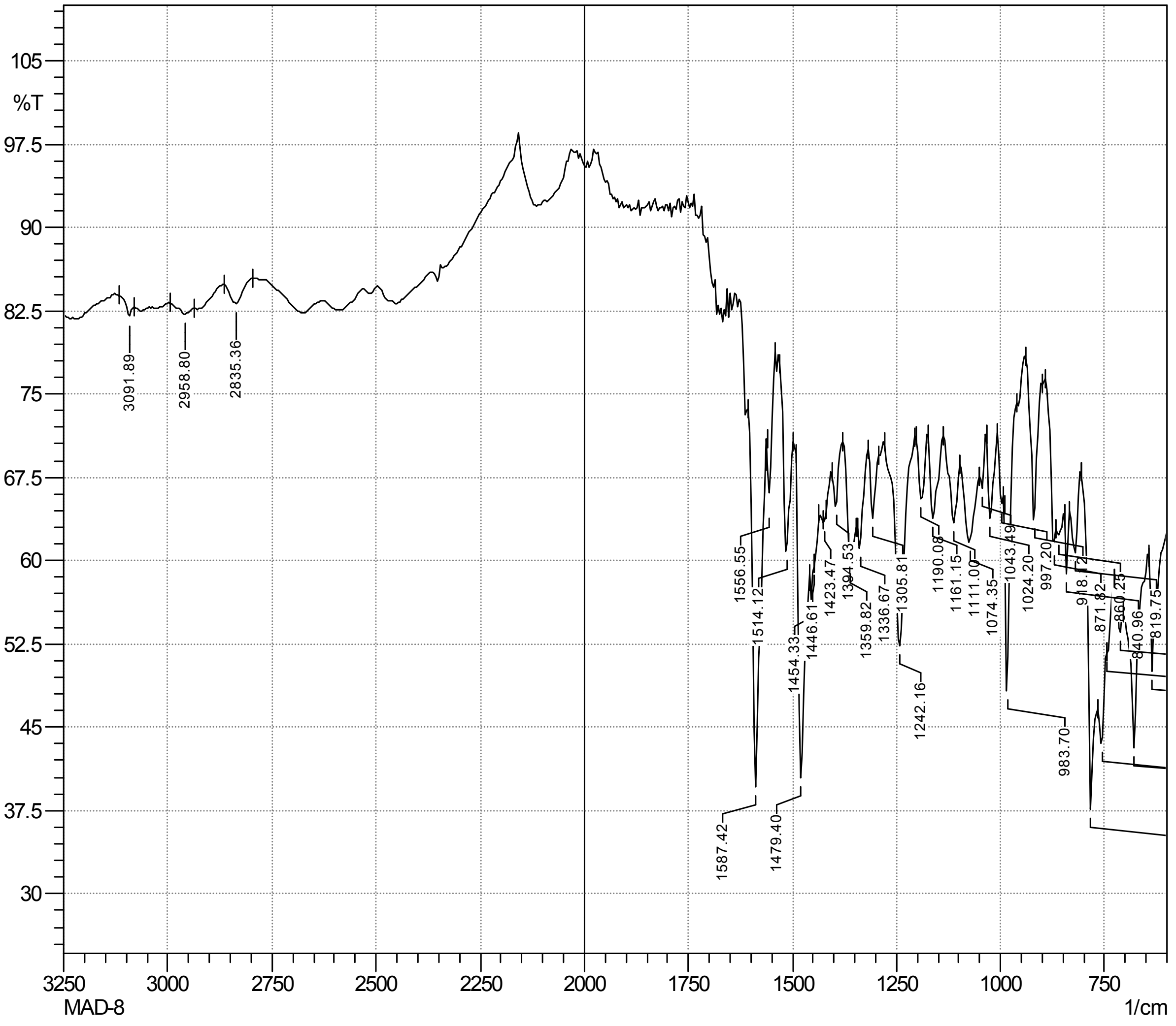

Figure S14. ^{1}H NMR spectrum of compound **2h**

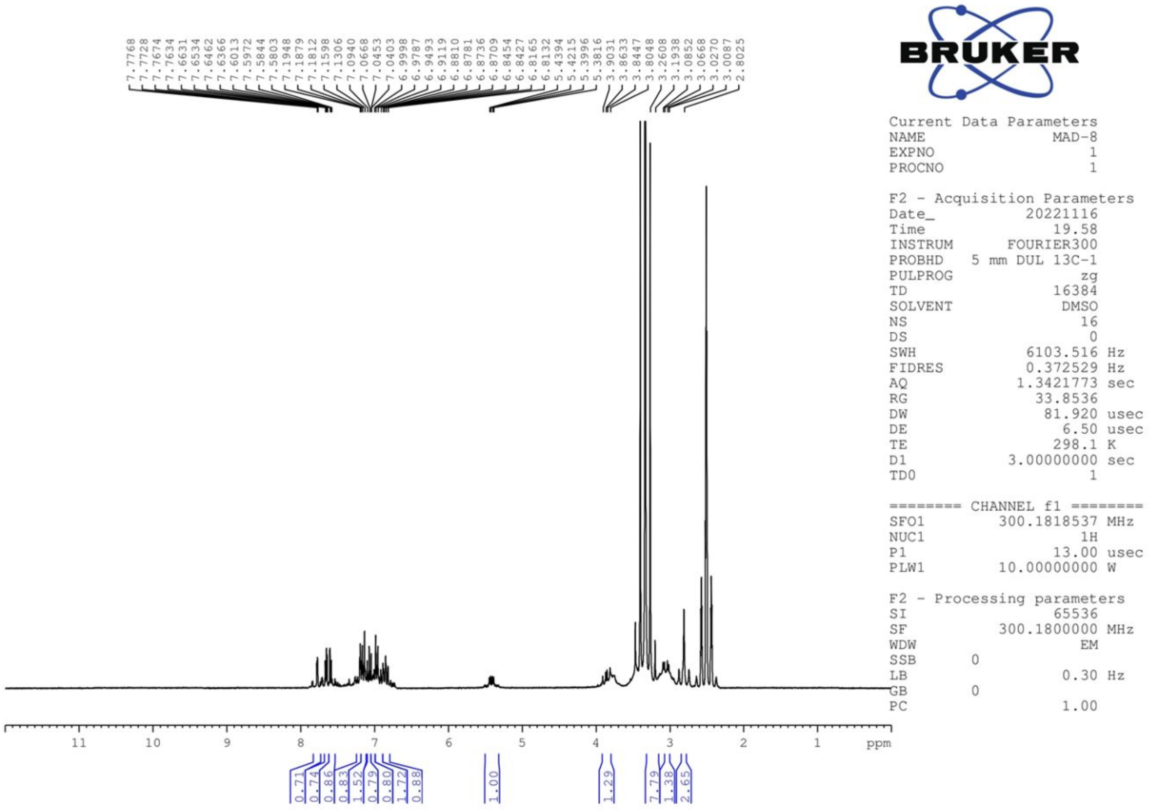

Figure S15. ^{13}C NMR spectrum of compound **2h**

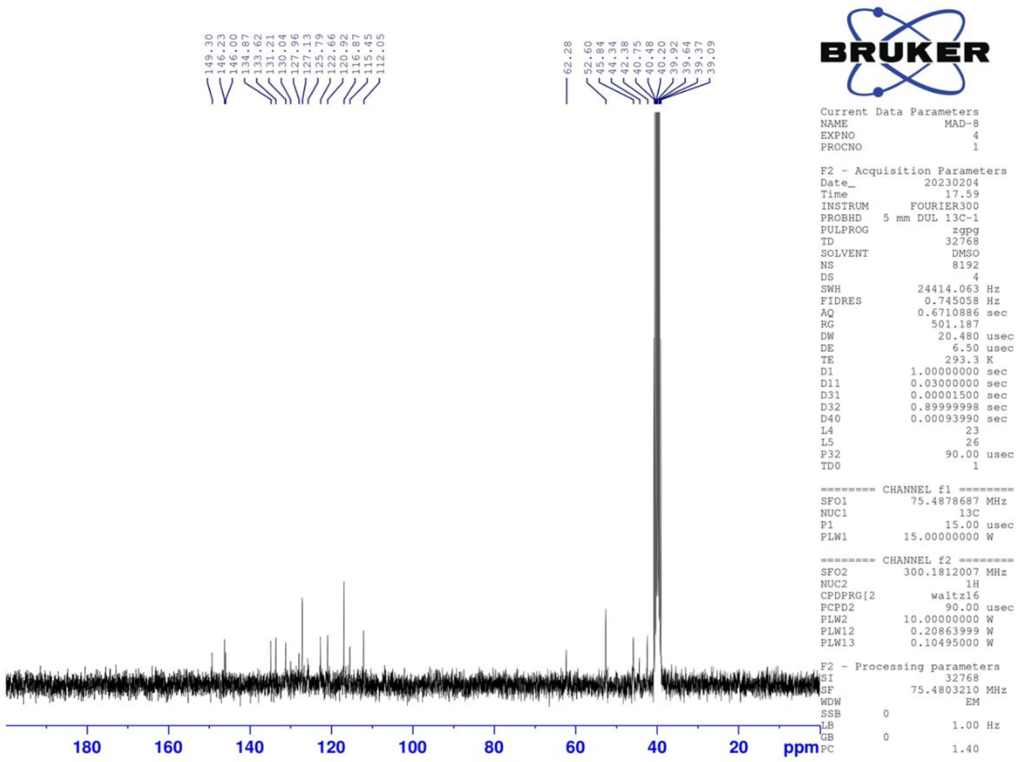

Figure S16. HRMS spectrum of compound **2h**

Formula Predictor Report - MAD-8_4.lcd

Data File: C:\LabSolutions\Data\Analiz\AOzdemir\MAD-8_4.lcd

Elmt	Val.	Min	Max	Elmt	Val.	Min	Max	Elmt	Val.	Min	Max	Elmt	Val.	Min	Max	Use Adduct
H	1	20	30	O	2	0	1	S	2	1	1	Ru	2	0	0	H
C	4	20	30	F	1	0	1	Cl	1	0	1	Pd	2	0	0	
N	3	0	5	P	3	0	0	Br	1	0	1	I	3	0	0	

Error Margin (ppm): 5
HC Ratio: unlimited
Max Isotopes: 3
MSn Iso RI (%): 10.00

DBE Range: 11.0 - 25.0
Apply N Rule: yes
Isotope RI (%): 1.00
MSn Logic Mode: AND

Electron Ions: odd
Use MSn Info: yes
Isotope Res: 9000
Max Results: 50

Event#: 1 MS(E+) Ret. Time : 3.920 -> 3.920 Scan# : 589 -> 589

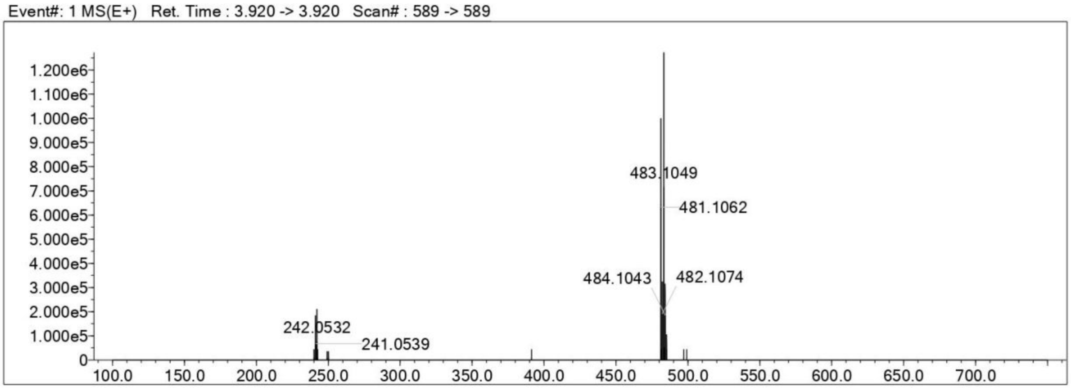

Measured region for 481.1062 m/z

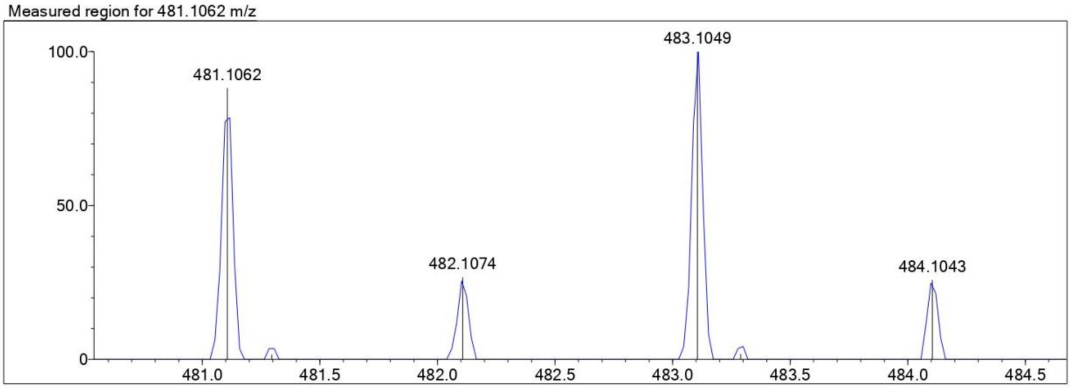

C24 H25 N4 S Br [M+H]+ : Predicted region for 481.1056 m/z

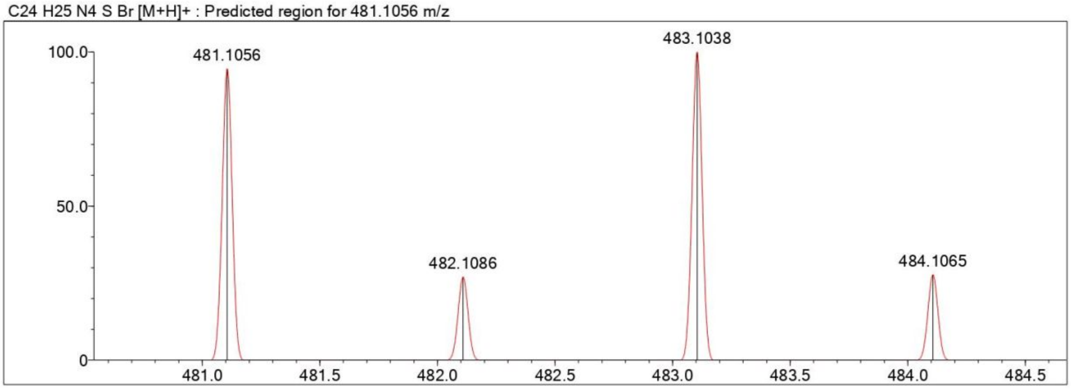

Rank	Score	Formula (M)	Ion	Meas. m/z	Pred. m/z	Df. (mDa)	Df. (ppm)	Iso	DBE
1	65.40	C24 H25 N4 S Br	[M+H]+	481.1062	481.1056	0.6	1.25	65.81	14.0
